# A weak allele of *TGW5* enables greater seed propagation and efficient size-based seed sorting for hybrid rice production

**DOI:** 10.1016/j.xplc.2024.100811

**Published:** 2024-01-11

**Authors:** Jiezheng Ying, Yaobing Qin, Fengyong Zhang, Liu Duan, Peng Cheng, Man Yin, Yifeng Wang, Xiaohong Tong, Jie Huang, Zhiyong Li, Xianjun Song, Jian Zhang

**Affiliations:** 1 State Key Lab of Rice Biology and Breeding, China National Rice Research Institute, Hangzhou 311400, China; 2 State Key Laboratory of Biocatalysis and Enzyme Engineering, School of Life Sciences, Hubei University, Wuhan 430062, China; 3 Key Laboratory of Plant Molecular Physiology, Institute of Botany, the Chinese Academy of Sciences, Beijing 100093, China

Dear Editors,

## Main text

**Figure 1:**
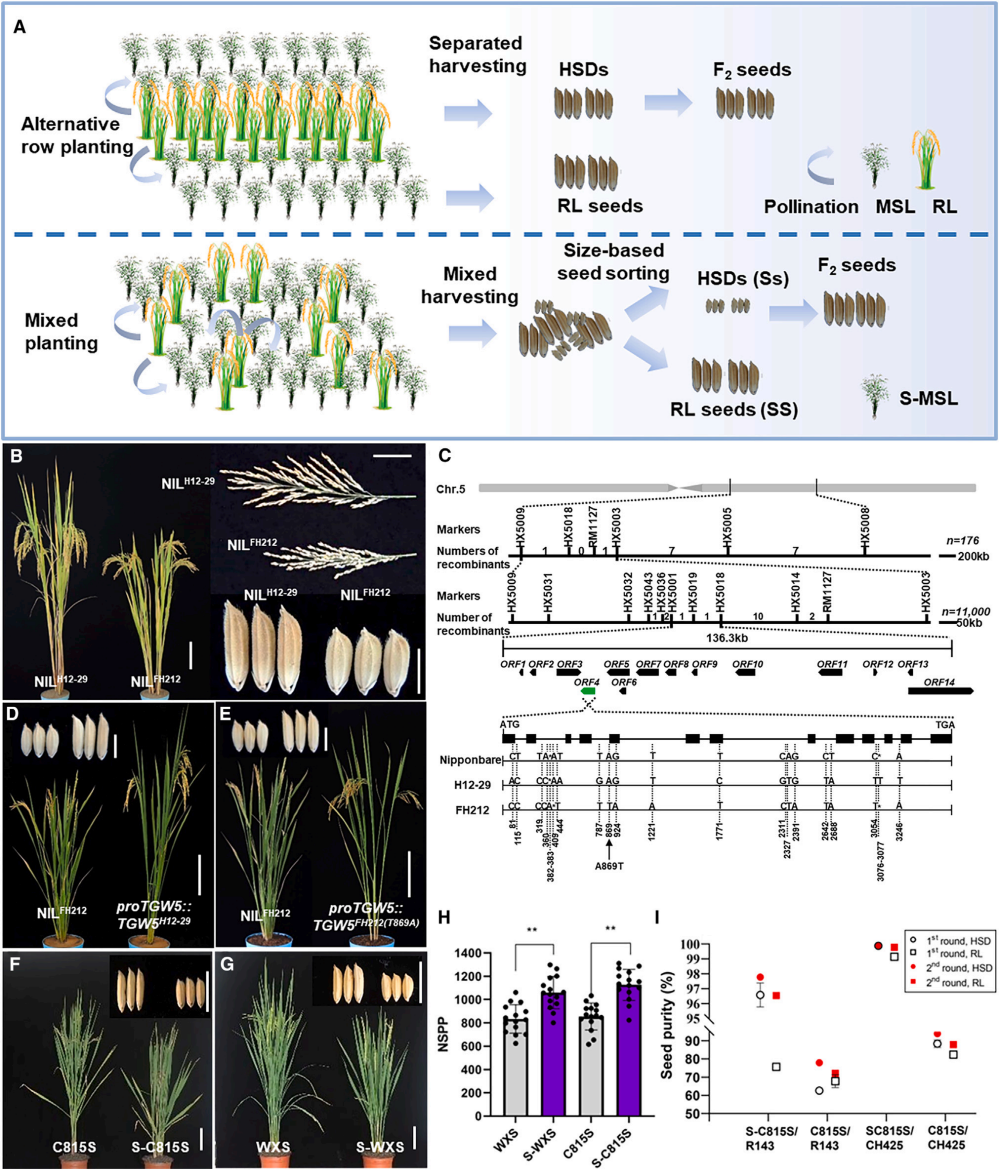
Cloning of *TGW5*, which controls grain size, and its use in mechanized production of rice hybrid seeds. **(A)** Flow model of HSD production using conventional alternative-row planting and S-MSL-based mixed-planting strategies. **(B)** Plant, panicle, and grain morphologies of the NIL lines. Plant, panicle, and grain scale bars represent 20 cm, 5 cm, and 5 mm, respectively. **(C)** Map-based cloning and molecular characterization of *TGW5*. **(D** **and E)** A genetic complementation test using *proTGW5::TGW5*^*H12-29*^ and *proTGW5::TGW5*^*FH212(T869A)*^ constructs. Scale bars for plants and grains represent 20 cm and 5 mm, respectively. **(F** **and G)** Plant and grain morphologies of C815S and S-C815S **(F)** and WXS and S-WXS **(G)**. Scale bars for plants and grains represent 12 cm and 10 mm, respectively. **(H)** Number of seeds per plant in the HSD production experiments. MSLs and S-MSLs were sterile and pollinated by the RL lines. ∗∗*P* < 0.01, Student’s *t*-test. **(I)** Seed purities of various seeds after sorting with an alveolar cylinder.

Heterosis utilization is an effective way to improve crop yield. Hybrid rice typically out-yield inbred rice varieties by 10% and show better stress resistance, and they have been widely adopted in Asian countries since the 1980s (Cheng et al., 2007). To produce rice F_1_ hybrid seeds (HSDs), male sterile lines (MSLs) are grown side by side with restorer lines (RLs) in order to receive the RL pollen. This seed production system faces challenges in maintaining the seed purity of the HSDs owing to the physical proximity of MSL and RL plants in the field. Traditionally, MSLs and RLs are planted in alternate rows to enable physical separation of seeds during harvesting. However, the complex field workflow and intensive workforce requirements significantly raise the cost of this production system. Alternatively, MSLs and RLs may be mixed planted, harvested, and subjected to post-harvest seed sorting, which is less expensive and more feasible for mechanized cultivation. Various post-harvest seed-sorting technologies have been invented based on female sterility of the RL (Xia et al., 2019), herbicide resistance (Fu et al., 2001), and morphological markers like the husk color of the MSLs (Dai, 1996) or the difference in seed size or weight between MSLs and RLs (Maruyama et al., 1991; Wu et al., 2021). When a small-seeded MSL (S-MSL) harboring a recessive seed-size locus (ss) is planted with a regular-seed-sized RL (SS), the HSDs (Ss) produced by the S-MSLs are small, owing to the maternal effect, and can easily be separated from the larger RL seeds using a mechanical sieve. The seed size of the F_2_ seeds is restored to an average level because their maternal genotype is Ss, thus maintaining the yield of F_2_ seeds at a desirable level (Figure 1A). However, S-MSLs are rarely used in production because of their time-consuming breeding methods, and there is an urgent need for novel genes applicable to S-MSL molecular breeding (Lv et al., 2023).

Using whole-genome sequencing and quantitative trait locus (QTL) sequencing, we mapped a major seed-weight QTL, *TGW5* (*Thousand-Grain-Weight 5*), from an RIL population of Hui 12-29 (H12-29; *Oryza sativa* L. ssp. *indica*, ♀) and Fuhui 212 (FH212; *Oryza sativa* L. ssp. *indica*, ♂) (Qin et al., 2018). Compared with those of NIL^H12-29^ (near isogenic line), all five internodes of NIL^FH212^ were shorter, resulting in a semi-dwarf phenotype (Figure 1B; Supplemental Figures 1 and 2A). NIL^FH212^ bore more panicles and seeds per plant than H12-29 (Figure 1B; Supplemental Figures 2B and 2C). More importantly, NIL^FH212^ showed a 25.4% decrease in grain length (GL) and an 8.6% increase in grain width (GW), resulting in a 37.6% decrease in TGW (Figure 1B; Supplemental Figures 2D–2G). The epidermal cell length and cell area of NIL^FH212^ were greater than those of H12-29, suggesting that *TGW5* controls seed size by regulating cell proliferation (Supplemental Figure 3). Seed size of the heterozygous NIL^FH212/H12-29^ was identical to that of NIL^H12-29^, indicating that *TGW5*^*FH212*^ is a complete recessive gene that confers a smaller seed size (Supplemental Figures 2E–2G).

*TGW5* was fine-mapped by map-based cloning to a 136.3-kb region of chromosome 5, which spanned 13 full open reading frames (ORFs). ORF4 (LOC_Os05g26890), which encodes a G protein α subunit, has previously been reported as a *D1* that controls plant height and seed size (Figure 1C), although the phenotypes of the reported *d1* were much more severe than those of NIL^FH212^ (Oki et al., 2009). Sanger sequencing of *TGW5* identified 10 polymorphisms in the introns and three synonymous SNPs in the exons, including an A869T SNP located in the conserved splice site at the 5′ end of the fifth exon (Figure 1C). RT–PCR and sequencing revealed that NIL^H12-29^ contains only a single transcript, whereas NIL^FH212^ harbors three transcript variants (Supplemental Figure 4A). Compared with the cDNA from H12-29 and Nipponbare, *TGW5-FH1* (the first transcript variant from FH212) contains a 19-bp insertion and a 13-bp deletion at the splicing site, whereas *TGW5-FH2* harbors only the 13-bp deletion, making the two variants indistinguishable by agarose electrophoresis. *TGW5-FH3* retains the fourth intron and is thus larger in size (Supplemental Figure 4B). In terms of their amino acid sequences, both TGW5-FH2 and TGW5-FH3 show premature termination, but the TGW5-FH1 protein is only slightly modified, with four variant residues and a two-residue insertion that may impair a conserved alpha-helix structure in the protein (Supplemental Figures 4C–4E). The CRISPR–Cas9-derived *TGW5* mutants *crj-01* and *crj-40* in the FH212 background showed a more severe dwarf phenotype and smaller grains than FH212, indicating that *TGW5*^*FH212*^ is partially functional (Supplemental Figure 5). Constructs *proTGW5::TGW5*^*H12-29*^ and *proTGW5::TGW5*^*FH212(T869A)*^ were introduced into NIL^FH212^ and fully rescued its plant height and seed size to NIL^H12-29^ levels (Figures 1D and 1E). We therefore concluded that *TGW5*^*FH212*^ is a new weak allele of *D1*. The A869T SNP in *TGW5*^*FH212*^ impairs mRNA splicing to produce a partially functional protein, TGW5-FH1, leading to a mild dwarf phenotype and a more minor seed phenotype in NIL^FH212^.

Given the significant effect of recessive *TGW5*^*FH212*^ on seed size, we developed two S-MSLs, S-C815S and S-WXS, by backcross breeding in two elite temperature-sensitive MSLs, C815S and WuxiangS (WXS), respectively (Figures 1F and 1G; Supplemental Figure 6A). In terms of key MSL features, the S-MSLs showed fertility transition temperatures similar to those of their corresponding MSLs (Supplemental Table 1) but a ∼20% reduction in stigma exertion rate compared with the MSLs (Supplemental Figures 6B–6D). At a fertile temperature, the S-MSLs had lower GL and TGW but slightly higher GW than their corresponding MSLs (Supplemental Figure 2). Notably, despite their somewhat lower absolute yield by weight, S-C815S and S-WXS set 31.31% and 27.82% more seeds per plant than C815S and WXS, respectively, mainly owing to their increased panicle numbers and spikelet density per panicle (Figure 1H; Supplemental Figures 2B–2D).

We next grew C815S and S-C815S at a sterile temperature and crossed them with the elite RLs R143 and CH425, respectively, to produce HSDs under agricultural conditions. The seed setting rates of C815S/CH425 and S-C815S/CH425 were 45.03% ± 3.04% and 38.15% ± 2.95%, respectively. Similarly, the seed setting rate of S-C815S/R143 (36.72% ± 2.78%) was slightly lower than that of C815S/R143 (40.75% ± 3.72%), possibly because of the lower stigma exertion rate of the S-MSLs. Interestingly, S-C815S/R143 and S-C815S/CH425 produced 12.79% and 10.84% more seeds than C815S/R143 and C815S/CH425, respectively, suggesting that the S-MSLs enable significantly higher seed propagation than the corresponding MSLs (Supplemental Figure 2C). An analysis of GL distribution indicated that the significant reduction of GL conferred by *TGW5*^*FH212*^ makes the S-MSL-derived HSDs separable from the RL seeds (Supplemental Figure 7). Indeed, S-C815S/CH425 HSDs (GL 6.58 ± 0.04 mm) and CH425 seeds (GL 10.72 ± 0.06 mm) could easily be sorted to reach an HSD purity of 99.87 ± 0.06% by one round of sorting with industrial alveolar cylinder equipment (Figure 1I; Supplemental Video 1). Moreover, the S-C815S/R143 HSDs (GL 6.61 ± 0.04 mm) and R143 seeds (GL 9.91 ± 0.05 mm) could also be effectively sorted to reach 96.56% ± 0.80% HSD seed purity (Figure 1I; Supplemental Video 2). By contrast, the regular MSL-derived HSDs and their corresponding RLs were not well separated, even after two rounds of sorting (Figure 1I; Supplemental Videos 3 and 4). This result clearly demonstrated that effective seed sorting of S-MSL-derived HSDs from RL seeds is highly feasible.

Finally, we evaluated the major agronomic traits of the F_1_ plants of C815S/R143, S-C815S/R143, C815S/CH425, and S-C815S/CH425 in the paddy field. The hybrid lines derived from MSLs and from their corresponding S-MSLs had almost identical phenotypes, indicating that the use of *TGW5* in the S-MSLs had no negative effect on heterosis of the F_1_ plants (Supplemental Figure 2).

In conclusion, we cloned a complete recessive seed size gene, *TGW5^FH212,^* that encodes a weak allele of *D1*. Introduction of *TGW5*^*FH212*^ converted regular MSLs into S-MSLs with increased seed numbers and reduced seed size but no penalties in terms of F_1_ heterosis. Although the trade-off between seed size and seed number is unfavorable for yield in the breeding practice of inbred lines, the smaller seed size and greater seed numbers conferred by *TGW5*^*FH212*^ turn out to be triple bonuses for HSD production: (1) they enable production of more HSDs per plant; (2) they facilitate effective post-harvest, size-based seed sorting that is compatible with labor-saving mechanized cultivation, which is estimated to reduce field costs by 16.3% (Tang et al., 2020); and (3) they reduce HSD storage and transport costs because of the smaller HSD seed size. Given that the currently used commercial MSLs and RLs have very similar seed sizes, *TGW5*^*FH212*^ is a highly promising resource for S-MSL breeding and is expected to increase HSD propagation and cut the cost of HSD production through mechanized cultivation.

## Funding

This work was supported by the 10.13039/501100001809National Natural Science Foundation of China (grant nos. 32072050, U22A20456, and U20A2030), a special support plan for high-level talents in Zhejiang (grant no. 2022R52020), the 10.13039/501100004731Zhejiang Provincial Natural Science Foundation of China (grant no. LZ21C130001), the 10.13039/501100012166National Key R&D Program of China (2020YFE0202300), Open Project Funding of the 10.13039/501100019937State Key Laboratory of Biocatalysis and Enzyme Engineering, the CNRRI key research and development project (CNRRI-2020-01), and the ASTIP program of CAAS.

## Author contributions

J.Z., J.Y., and X.S. planned and designed the research; Y.Q., J.Y., F.Z., L.D., P.C., M.Y., Y.W., X.T., J.H., and Z.L. performed experiments; J.Z., Y.Q., J.Y., and F.Z. analyzed data; and J.Z. and J.Y. wrote the manuscript.

## Supplemental information

Document S2. Supplemental Tables 1 and 2

Supplemental Video 1. Sorting of S-C815S/CH425 HSDs and CH425 seeds through an alveolar cylinder CH425 seeds were stained with black ink to better visualize the seed-sorting results.3

Supplemental Video 2. Manual sorting of S-C815S/R143 HSDs and R143 seeds through a sieve R143 seeds were stained with red ink to better visualize the seed-sorting results.4

Supplemental Video 3. Sorting of C815S/CH425 HSDs and CH425 seeds through an alveolar cylinder HSDs were stained with green ink to better visualize the seed-sorting results.5

Supplemental Video 4. Sorting of C815S/R143 HSDs and R143 seeds through an alveolar cylinder R143 seeds and HSDs were stained with red and green inks, respectively, to better visualize the seed-sorting results.6

Document S2. Article plus supplemental information
