# Supplementary material for: A weak allele of TGW5 enables greater seed propagation and efficient size-based seed sorting for hybrid rice production
Source: Plant Commun. 2024 Jan 11;5(4):100811. doi: 10.1016/j.xplc.2024.100811 (PMC11009153; doi:10.1016/j.xplc.2024.100811)
Supplement: Document S1. Supplemental Figures 1–7 [file mmc1.pdf]

Plant Communications, Volume *5*

Supplemental information

A weak allele of *TGW5* enables greater seed propagation and efficient size-based seed sorting for hybrid rice production

Jiezheng Ying, Yaobing Qin, Fengyong Zhang, Liu Duan, Peng Cheng, Man Yin, Yifeng Wang, Xiaohong Tong, Jie Huang, Zhiyong Li, Xianjun Song, and Jian Zhang

Materials and Methods

Plant materials and field trials

To identify the *TGW5* gene, a large grain indica variety Hui 12-29 (H12-29) was crossed with a small grain *indica* variety Fuhui 212 (FH212) to develop advanced genetic populations. Recombinant plants in the F_8 generation with identical backgrounds but heterozygous *TGW5* region were selected to develop segregating populations for fine mapping. From one recombinant plant in F_{10} generation, we developed a pair of nearly isogenic lines for *TGW5*, $NIL^{H12-29}$and NIL^{FH212}, with isogenic homozygous background and a small homozygous chromosomal segment of H12-29 and FH212 containing *TGW5*, respectively. To develop thermo-sensitive genic male sterile lines with small grain size, NIL^{FH212} was crossed with two commercial thermo-sensitive male sterile lines, C815S and WuxiangS. After successive backcrosses with C815S and WuxiangS and marker assistant selections, two thermo-sensitive male sterile lines with small grain size in BC_3F_5 generation, S-C815S and S-WXS, were developed and characterized by *de novo* genome sequencing.

Rice materials were planted with a spacing of 16.6 cm × 26.7 cm in the experimental stations of China National Rice Research Institute in Hangzhou (119°57' E, 30°05' N) and Lingshui (109°45' E, 18°22' N). Eighteen plants for each line were planted in 3 rows, and the 4 plants in the middle of the row were harvested for agronomic traits characterization. For HSD production, MSLs and RLs were grown in separate rows with a ratio of 7:2 (every 7 rows of MSLs neighbored by 2 rows of RLs). Field management followed the local agricultural practice in production.

Fine mapping and candidate gene analysis

Segregating populations containing about 11,000 plants in the F_9 generation were used to screen the recombinants in the target region around *TGW5*. We developed molecular markers based on 30× genome re-sequencing of the parental lines and detected 17 recombinants in the target region containing *TGW5*. The F_{10} progeny

derived from the recombinant plants were used to screen homozygous recombination products and measure the grain size, including grain length, width, and 1000-grain weight. The candidate *TGW5* genes from H12-29 and FH212 genomic DNA were PCR amplified and sequenced. The sequences of the primers are provided in Table S2.

RNA extraction and cDNA isolation

Total RNA was extracted from various tissues of the parental lines, H12-29 and FH212, using RNeasy Plant Mini Kit (QIAGEN, Germantown, MD, USA) and was converted into cDNA with ReverTra Ace qPCR RT Master Mix with gDNA Remover (TOYOBO, Shanghai, China). Full-length *TGW5* cDNA was amplified from the first-strand cDNA with KOD-Plus-Neo Kit (TOYOBO, Shanghai, China) and sequenced.

Vector construction and transgenic analysis

We developed EH2 primers with restriction enzyme cleavage sites of *Eco*R I and *Hin*d III gDNA to amplify gDNA sequences of *TGW5* containing 2,000 bp promoter regions, full-length ORFs, and 500 bp terminator sequences from H12-29 and FH212 genomic DNA using KOD FX Neo (TOYOBO, Shanghai, China), respectively. Both *TGW5* gDNA sequences were sub-cloned into the vector pCambia 1301. We further developed XD1 primers to create SNP mutation T869A in the *TGW5* gDNA sequence of FH212 in the pCambia 1301 using the Fast Mutagenesis System (TransGen, Beijing, China). *TGW5* gene in FH212 was knocked out using the CRISPR/Cas9 system according to a previously described protocol. The constructs containing the *TGW5* gDNA sequence of H12-29 and the T869A point mutation *TGW5* gDNA sequence of FH212 were used for the complementation test and mutation validation. The resulting constructs were introduced into *Agrobacterium tumefaciens* strain EHA105 and transferred into NIL^{FH212}. The sequences of the primers are provided in Table S2.

Measurement of agronomic traits

Rice grains collected from the paddy field were first air-dried, then dried at 37℃ in the oven for 24 hours. Four traits, including plant height, number of panicles per plant (NPP), seeds per plant, grain yield per plant, and stigma exertion rate were measured manually. The filled grains were chosen to identify the grain traits, including grain length (GL), grain width (GW), and 1000-grain weight (TGW) using an SC-A seed counting and grain weighting device (Wanshen Ltd, Hangzhou, China).

Histological observation

To determine the cell size, cell length, and cell width, spikelet hulls of mature grain of NILs (*TGW5*) and transgenic plants were sputter-coated with platinum and observed using a scanning electron microscope (S-4800; Hitachi, Japan). Cell size, length, width, and cell number in the outer parenchyma layer of the spikelet hulls were analyzed using ImageJ software.

Protein sequence analysis

Multiple alignments of amino acid sequences of TGW5 were conducted online (https://www.ebi.zc.uk/Tools/msa/clustalo) and were described using the Sequence Manipulation Suite (Li et al., 2016). SWISS-MODEL was used in the homology modeling of TGW5 protein conformation. Protein structures corresponding to different transcripts of the *TGW5* gene were analyzed with Swiss-Pdb Viewer.

GL-based seed sorting

The HSD and RL seeds were mixed and sorted through an alveolar cylinder (Westrup LA-T, Denmark) with an alveolar diameter of 8.5 mm and a cylinder rotating speed in level 1. The vibration frequency was set to level 1 for S-C815S/R143 sorting and level 2 for the other samples.

Figure S1. Comparison of the rice stem length between the NILs. (A) The internodes of $NIL^{H12\text{-}29}$ and NIL^{FH212}. Bar, 20 cm. (B) Internode lengths relative to the total length of the stem. (C) The percentage of internode length relative to the total internode length. White arrows indicate the nodes.

Figure S2. The major agronomic traits of the genetic materials.
(A) Plant height. (B) Panicles per plant. (C) Seeds per plant. (D) Grain yield per plant. (E) Grain length. (F) Grain width. (G) Thousand-grain weight. For data in grey, pink, and purple bars, plants were grown in Hangzhou, Zhejiang, China (119°57' E, 30°05' N), where the average temperature was above 25℃ during the early panicle differentiation stage at the end of July 2023. All the MSLs were sterile and cross-pollinated by RLs, while inbred lines were fully fertile. For data in orange and green bars, plants were grown in Lingshui, Hainan, China (109°45' E, 18°22' N), where the average temperature was below 22℃ during the early panicle differentiation stage in the middle of February 2023. All the MSLs were fertile and self-pollinated. *, $P<0.05$; **, $P<0.01$ by *students*' t-test.

Figure S3. Cytological feature observations of exterior epidermal cells of the spikelet hulls of $NIL^{H12\text{-}29}$ and NIL^{FH212}. (A-B) Exterior epidermal cells of the lemma of $NIL^{H12\text{-}29}$ (A) and NIL^{FH212} (B), Bar, 100μm. (C) Comparison of cell length of the spikelet hulls of $NIL^{H12\text{-}29}$ and NIL^{FH212}. (D) Comparison of cell width of the spikelet hulls of $NIL^{H12\text{-}29}$ and NIL^{FH212}. (E) Comparison of cell size of the spikelet hulls of $NIL^{H12\text{-}29}$ and NIL^{FH212}. Scale bar, 100 μm. *, $P<0.05$, ****, $P<0.0001$.

Figure S4. Structural and sequence analysis of *TGW5*. (A) Electrophoresis of *TGW5* gDNA and cDNA PCR products from the parental lines H12-29 and FH212, FH1, FH2, and FH3 are the three transcripts of $TGW5^{FH212}$. (B) Schematic presentation of the variations on *TGW5* transcript. (C) Alignment of cDNA sequences of TGW5-FH variants. TGW5-FH2 and TGW5-FH3 are pre-matured at position 574 and 352, respectively. (D) Alignment of amino acid sequences of TGW5 between H12-29 and TGW5-FH1. Red box, amino acid variations for the impaired alpha helix structure in FH1. (E) Predicted three-dimensional structure of TGW5 based on the deduced amino acid sequence. The red circle indicates the mutation site of TGW5-FH1, potentially resulting in an impaired alpha helix structure.

Figure S5. Phenotypes and genotypes of CRISPR/Cas9-derived *TGW5* knock-out lines in FH212 background. (A-C) Plant (A), grain (B), and panicle (C) morphologies of wild-type FH212 and gene-edited lines. Bar, 10 cm in A, 1 cm in B, and 2 cm in C. (D) Sequence alignment of the targeted mutation site in *TGW5*. The PAM sequences are highlighted in yellow. - indicates base deletion; Red font indicates base insertions.

Figure S6. Schematic presentation of the genetic backgrounds and stigma exertion features of S-C815S and S-WXS.

(A) Genome *de novo* sequencing results showed that there are 5.71% and 4.78% of the donor chromosome segments in S-C815S (BC_3F_5) and S-WXS (BC_3F_5), respectively. The black bar indicates the genome fragment from donor FH212. (B-C) The stigma exertion features of C815S (B) and S-C815S (C). The embedded pictures on the bottom depict a zoomed-in view of the white frame at the top. The red triangle indicates florets with fully exerted stigmas. Scale bars, 5 mm. (J) Total percentage of the exerted stigma (TPES). ** indicates $P<0.01$ by *students*' t-test, n=50.

Figure S7. The grain length distribution frequencies of HSDs and RLs.

(A) The grain length distribution of R143 and HSDs. (B) The grain length distribution of CH425 and HSDs. All the data were randomly collected from over 500 grains of each line.

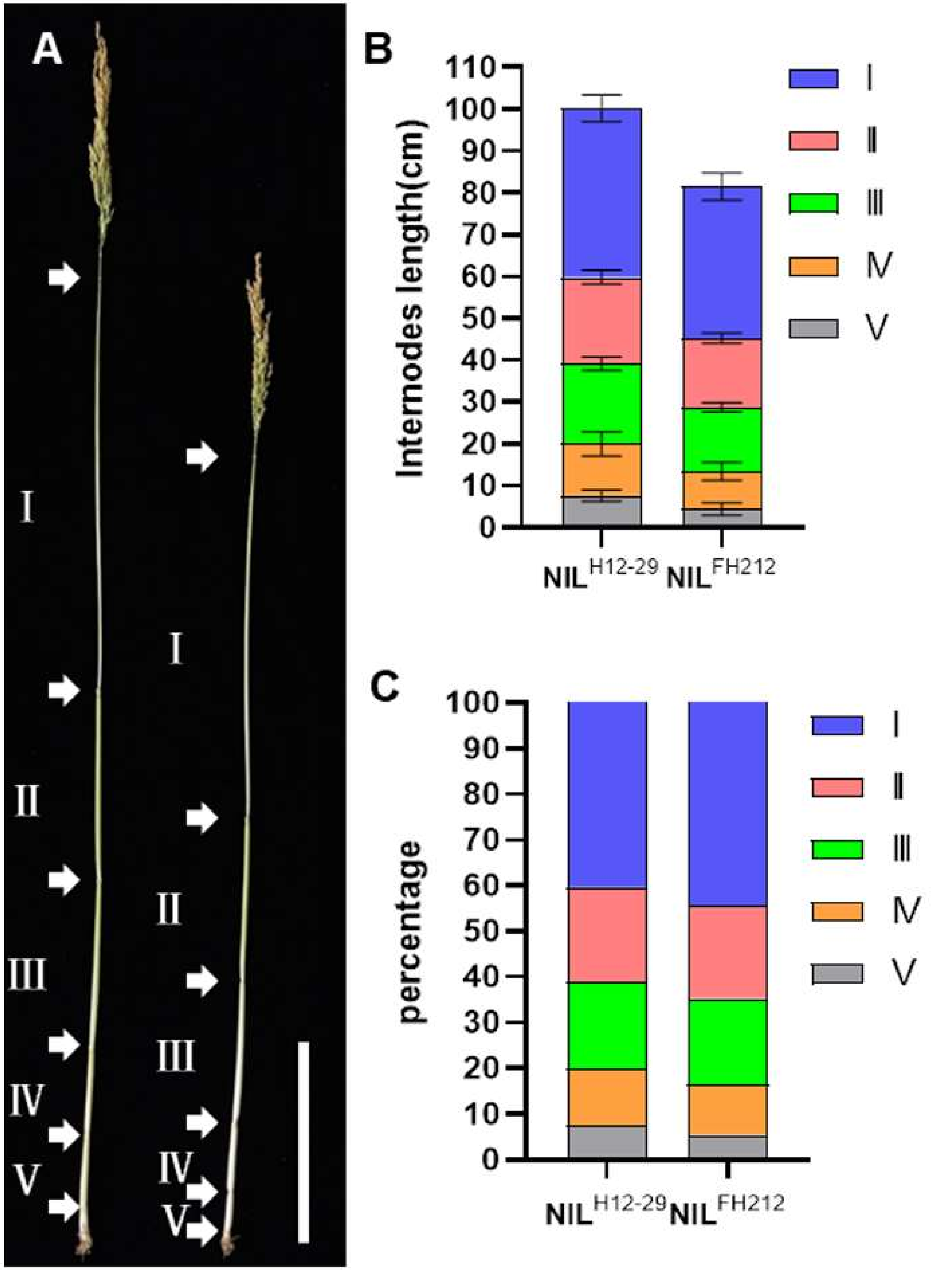
A
I
II
III
IV
V
I
II
III
IV
V
NIL^{H12-29}
NIL^{FH212}
B
Internodes length(cm)
110
100
90
80
70
60
50
40
30
20
10
0
NIL^{H12-29}
NIL^{FH212}
Ⅰ
Ⅱ
Ⅲ
Ⅳ
Ⅴ
C
percentage
100
90
80
70
60
50
40
30
20
10
0
NIL^{H12-29}
NIL^{FH212}
Ⅰ
Ⅱ
Ⅲ
Ⅳ
Ⅴ

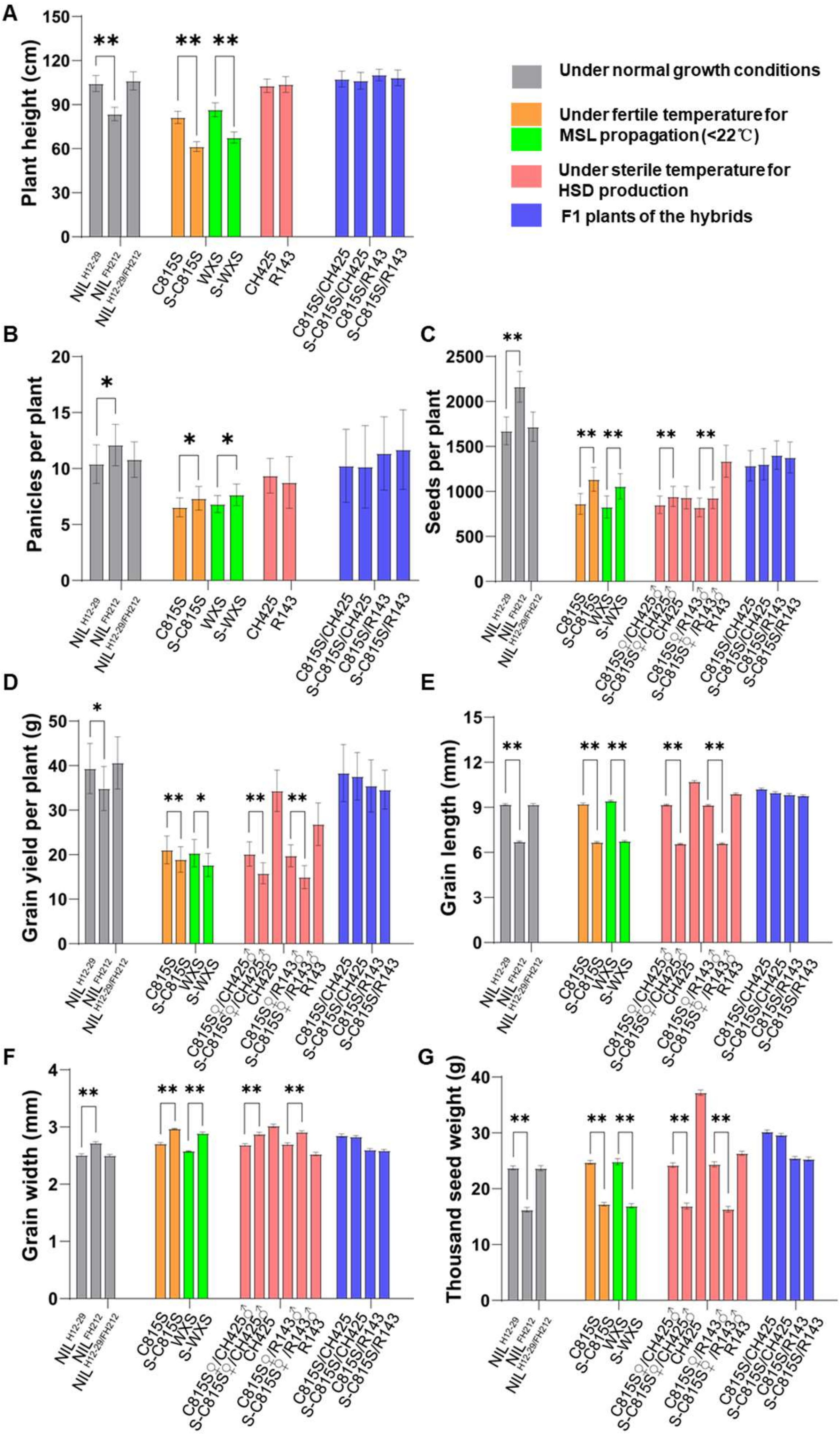
A
B
C
D
E
F
G
Under normal growth conditions
Under fertile temperature for MSL propagation (<22℃)
Under sterile temperature for HSD production
F1 plants of the hybrids
Plant height (cm)
Panicles per plant
Seeds per plant
Grain yield per plant (g)
Grain length (mm)
Grain width (mm)
Thousand seed weight (g)
NIL H12-29
NIL FH212
NIL H12-29/FH212
C815S
S-C815S
WXS
S-WXS
CH425
R143
C815S♀/CH425♂
S-C815S♀/CH425♂
C815S♀/R143♂
S-C815S♀/R143♂
C815S/CH425
S-C815S/CH425
C815S/R143
S-C815S/R143
**
*

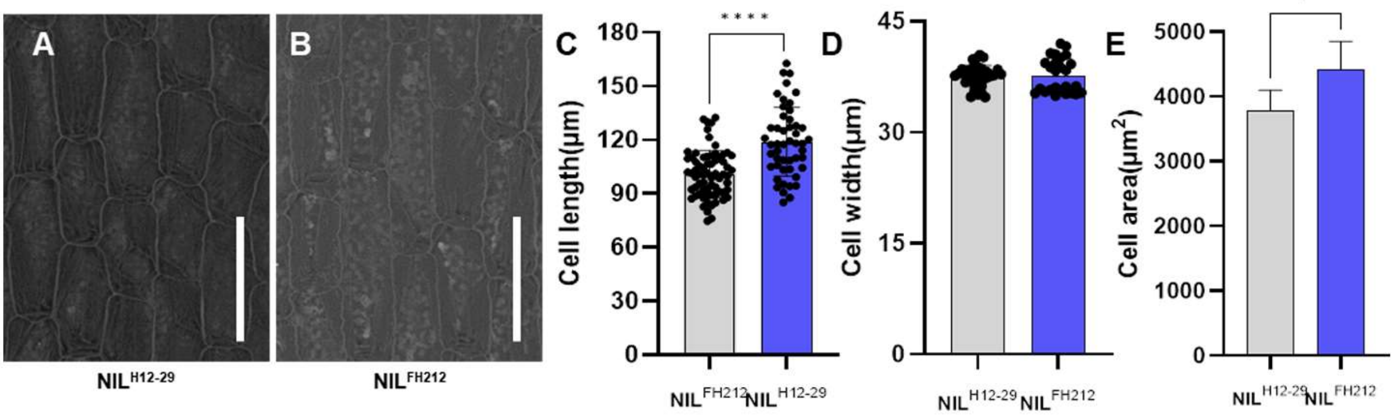
A
B
NIL^H12-29
NIL^FH212
C
Cell length(μm)
180
150
120
90
60
30
0
****
NIL^FH212 NIL^H12-29
D
Cell width(μm)
45
30
15
0
NIL^H12-29 NIL^FH212
E
Cell area(μm²)
5000
4000
3000
2000
1000
0
*
NIL^H12-29 NIL^FH212

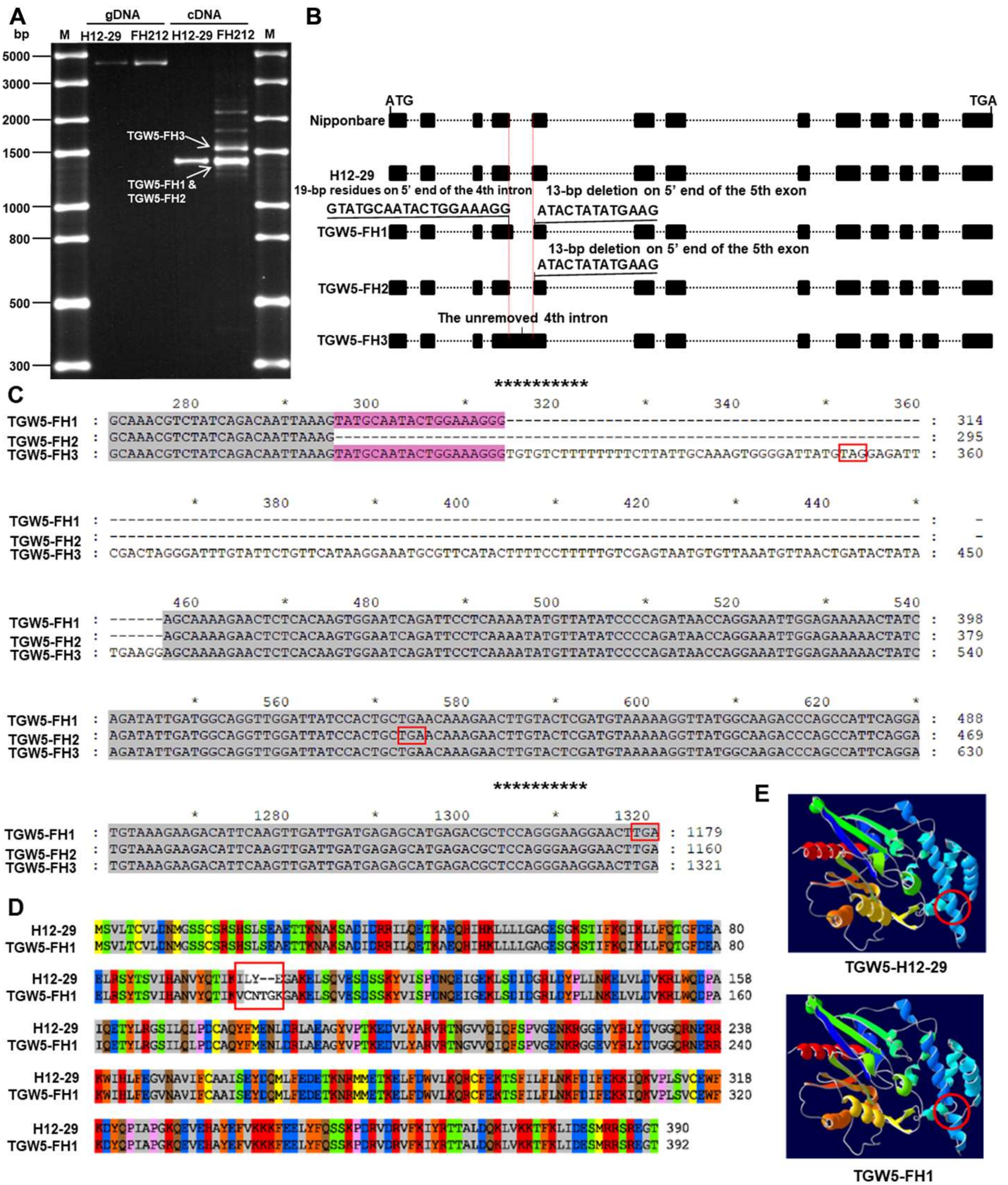
A
bp
M
gDNA
H12-29
FH212
cDNA
H12-29
FH212
M
5000
3000
2000
1500
1000
800
500
300
TGW5-FH3
TGW5-FH1 &
TGW5-FH2
B
ATG
TGA
Nipponbare
H12-29
19-bp residues on 5' end of the 4th intron
13-bp deletion on 5' end of the 5th exon
GTATGCAATACTGGAAAGG
ATACTATATGAAG
TGW5-FH1
13-bp deletion on 5' end of the 5th exon
ATACTATATGAAG
TGW5-FH2
The unremoved 4th intron
TGW5-FH3
C
TGW5-FH1
TGW5-FH2
TGW5-FH3
D
H12-29
TGW5-FH1
E
TGW5-H12-29
TGW5-FH1

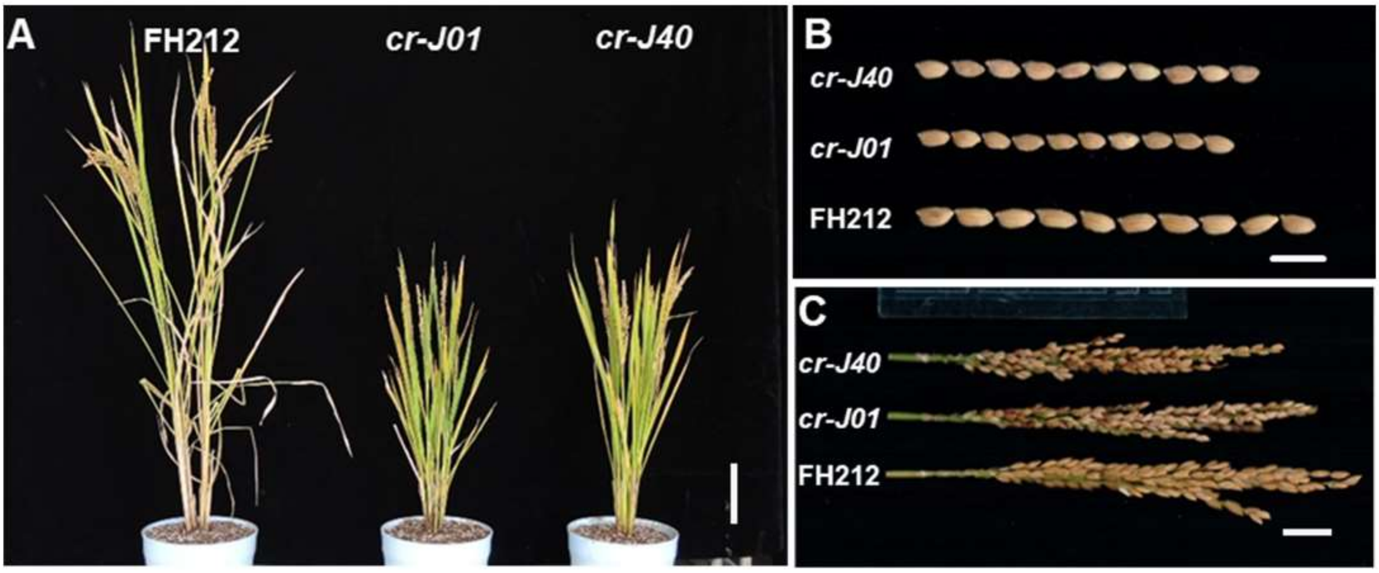

D

FH212 TAGGAGGCCAGAGGAATGAGAGGAGAAAGTGGATTCATCTTTTTGA

Cr-J01 TAGGAGGCCAGAGGAATGAGAG- - GAAAGTGGATTCATCTTTTTGA

Cr-J40 TAGGAGGCCAGAGGAATGAGAGGAGACACAAAAAGTGGATTCATCTTTTTGA

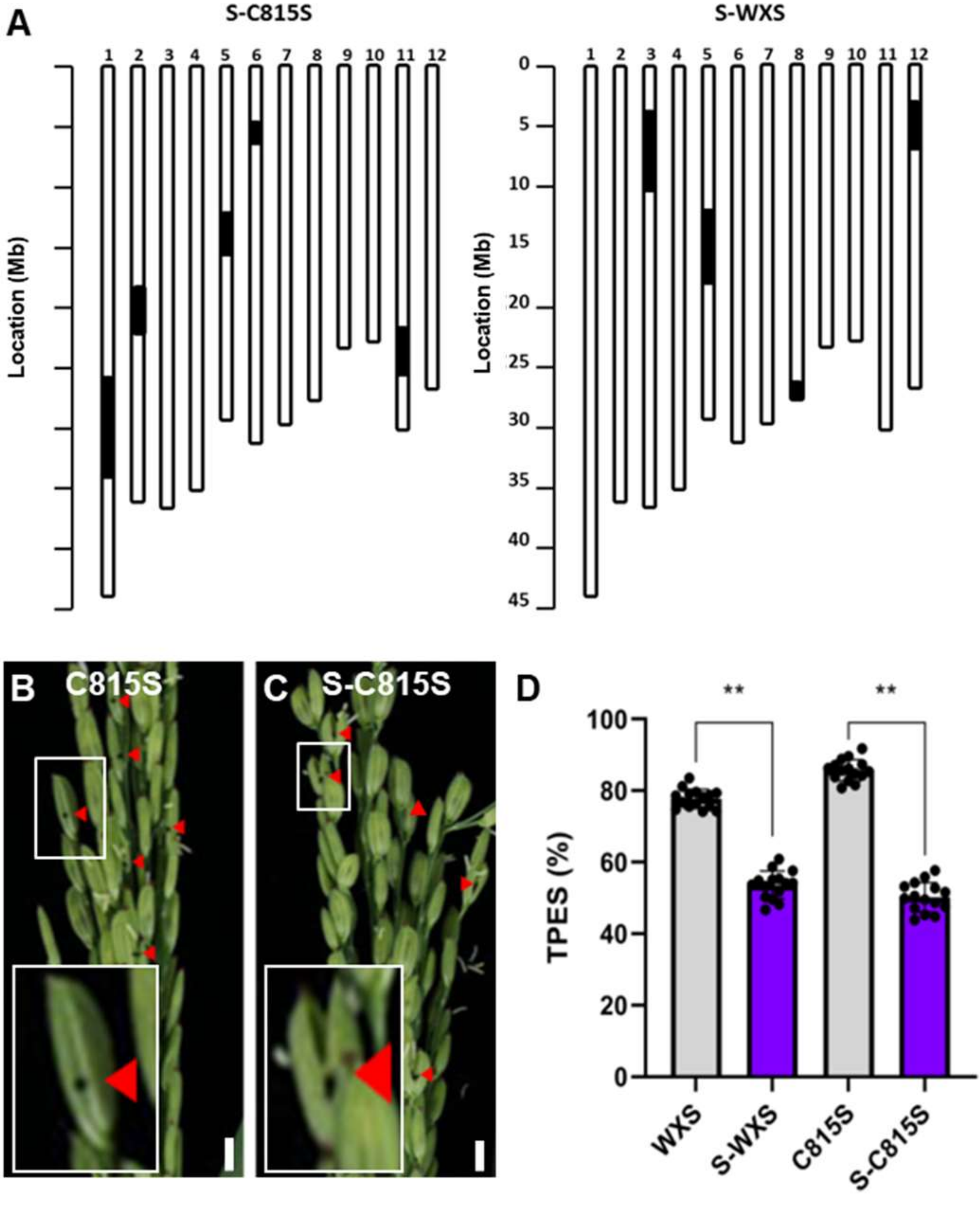
A
S-C815S
S-WXS
1
2
3
4
5
6
7
8
9
10
11
12
0
5
10
15
20
25
30
35
40
45
Location (Mb)
B
C815S
C
S-C815S
D
**
TPES (%)
0
20
40
60
80
100
WXS
S-WXS
C815S
S-C815S

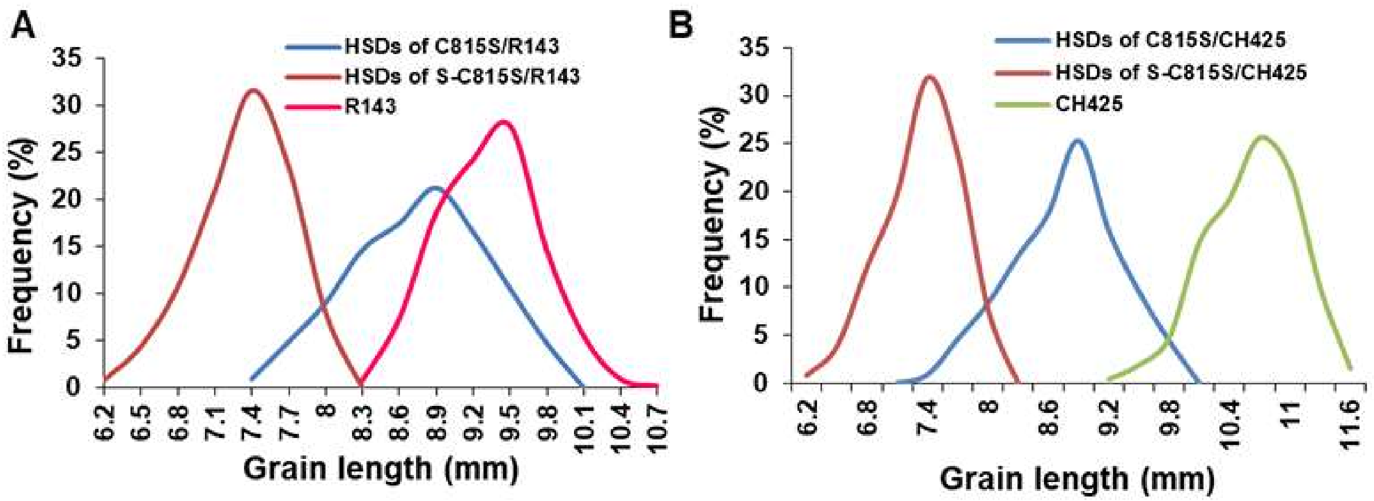

A
HSDs of C815S/R143
HSDs of S-C815S/R143
R143
Frequency (%)
Grain length (mm)
B
HSDs of C815S/CH425
HSDs of S-C815S/CH425
CH425
Frequency (%)
Grain length (mm)
