# Supplementary material for: A weak allele of TGW5 enables greater seed propagation and efficient size-based seed sorting for hybrid rice production
Source: Plant Commun. 2024 Jan 11;5(4):100811. doi: 10.1016/j.xplc.2024.100811 (PMC11009153; doi:10.1016/j.xplc.2024.100811)
Supplement: Document S2. Supplemental Tables 1 and 2 [file mmc2.pdf]

Table S1. Pollen fertility of the MSLs and S-MSLs under different temperatures

ID	Pollen fertility by I_2-KI staining (%)			
	20.5℃	21.5℃	22.5℃	23.5℃
C815S	41.74±6.32	13.48±2.13	0±0	0±0
S-C815S	43.86±5.43	14.65±1.85	0±0	0±0
WXS	37.75±8.16	17.32±3.13	0.56±0.16	0±0
S-WXS	34.58±6.11	16.87±4.21	0.77±0.18	0±0

The plants in early panicle differentiation stage were moved to grow in a growth chamber with the set temperature and day length of 14h. Over 25 florets on 5 plants of each line were collected from the main panicle for pollen fertility analysis by I_2-KI staining method.

Table S2. The sequence of primers used in this study.

Name	Forward primer (5' to 3')	Reverse primer (5' to 3')	Enzyme	Usage
HX5031	ACCGTGCTTGTTGTAGTCGTG	GAGAAAGGGATTTGGTGATGG		mapping
HX5032	ATATTTTGAAATGGAGGAAG	AGTACCGATAGGATGGAGA		mapping
HX5043	AACGAGGGTGTCAGCAAGA	ACAGCGAGGGTGGAAACTA		mapping
HX5036	CTTTCAGAGGCAGACAGAT	ATAATTGCTTGGACCACAT		mapping
HX5001	GGTCTCACCTCACAGAATACCGAAAG	ATAGCCGACTCCGCAACAAGGAC		mapping
HX5019	TATAGATGATTGGATGAGAAT	TTAGAACGAAGAAAGTAGTAA		mapping
HX5018	TCAGCATCGGAACAAATC	GAAGGGACGGACAGAAAC		mapping
HX5014	AGTTCTTGGAGGGCTTTATTC	TTGTGATAGTTGGCTCTGGAC		mapping
HX5009	AAACAAGAGCCAAAGAAGTCA	TTCCGCTACATCCAACAAATA		mapping
HX5003	CTCAGGAAGGTAGTCCGAGTCAT	CACAAGCCGTCAAGTTTATCACA		mapping
EH2	CCGGAATTCCAAACCCCGTTAAAGCC	CCCAAGCTTTCCCTGCCAACCGACA	EcoR I & Hind III	Amplification of *TGW5* gene
XD1	GTGTTAAATGTTAACAGATACTATATGAAGG	TGTTAACATTTAACACATTACTCGACAAAAA		Vector construction of SNP mutation
