# Supplementary material for: A weak allele of TGW5 enables greater seed propagation and efficient size-based seed sorting for hybrid rice production
Source: Plant Commun. 2024 Jan 11;5(4):100811. doi: 10.1016/j.xplc.2024.100811 (PMC11009153; doi:10.1016/j.xplc.2024.100811)
Supplement: Document S2. Article plus supplemental information [file mmc7.pdf]

Plant Communications
Correspondence

CellPress
Partner Journal

A weak allele of *TGW5* enables greater seed propagation and efficient size-based seed sorting for hybrid rice production

Dear Editors,

Heterosis utilization is an effective way to improve crop yield. Hybrid rice typically out-yield inbred rice varieties by 10% and show better stress resistance, and they have been widely adopted in Asian countries since the 1980s (Cheng et al., 2007). To produce rice F_1 hybrid seeds (HSDs), male sterile lines (MSLs) are grown side by side with restorer lines (RLs) in order to receive the RL pollen. This seed production system faces challenges in maintaining the seed purity of the HSDs owing to the physical proximity of MSL and RL plants in the field. Traditionally, MSLs and RLs are planted in alternate rows to enable physical separation of seeds during harvesting. However, the complex field workflow and intensive workforce requirements significantly raise the cost of this production system. Alternatively, MSLs and RLs may be mixed planted, harvested, and subjected to post-harvest seed sorting, which is less expensive and more feasible for mechanized cultivation. Various post-harvest seed-sorting technologies have been invented based on female sterility of the RL (Xia et al., 2019), herbicide resistance (Fu et al., 2001), and morphological markers like the husk color of the MSLs (Dai, 1996) or the difference in seed size or weight between MSLs and RLs (Maruyama et al., 1991; Wu et al., 2021). When a small-seeded MSL (S-MSL) harboring a recessive seed-size locus (ss) is planted with a regular-seed-sized RL (SS), the HSDs (Ss) produced by the S-MSLs are small, owing to the maternal effect, and can easily be separated from the larger RL seeds using a mechanical sieve. The seed size of the F_2 seeds is restored to an average level because their maternal genotype is Ss, thus maintaining the yield of F_2 seeds at a desirable level (Figure 1A). However, S-MSLs are rarely used in production because of their time-consuming breeding methods, and there is an urgent need for novel genes applicable to S-MSL molecular breeding (Lv et al., 2023).

Using whole-genome sequencing and quantitative trait locus (QTL) sequencing, we mapped a major seed-weight QTL, *TGW5* (*Thousand-Grain-Weight 5*), from an RIL population of Hui 12-29 (H12-29; *Oryza sativa* L. ssp. *indica*, ♀) and Fuhui 212 (FH212; *Oryza sativa* L. ssp. *indica*, ♂) (Qin et al., 2018). Compared with those of $NIL^{H12\text{-}29}$ (near isogenic line), all five internodes of NIL^{FH212} were shorter, resulting in a semi-dwarf phenotype (Figure 1B; Supplemental Figures 1 and 2A). NIL^{FH212} bore more panicles and seeds per plant than H12-29 (Figure 1B; Supplemental Figures 2B and 2C). More importantly, NIL^{FH212} showed a 25.4% decrease in grain length (GL) and an 8.6% increase in grain width (GW), resulting in a 37.6% decrease in TGW (Figure 1B; Supplemental Figures 2D–2G). The epidermal cell length and cell area of NIL^{FH212} were greater than those of H12-29, suggesting that *TGW5* controls seed size by regulating cell proliferation (Supplemental Figure 3). Seed size of the heterozygous $NIL^{FH212/H12\text{-}29}$ was identical to that of $NIL^{H12\text{-}29}$, indicating that $TGW5^{FH212}$ is a complete recessive gene that confers a smaller seed size (Supplemental Figures 2E–2G).

TGW5 was fine-mapped by map-based cloning to a 136.3-kb region of chromosome 5, which spanned 13 full open reading frames (ORFs). ORF4 (LOC_Os05g26890), which encodes a G protein α subunit, has previously been reported as a *D1* that controls plant height and seed size (Figure 1C), although the phenotypes of the reported *d1* were much more severe than those of NIL^{FH212} (Oki et al., 2009). Sanger sequencing of *TGW5* identified 10 polymorphisms in the introns and three synonymous SNPs in the exons, including an A869T SNP located in the conserved splice site at the 5′ end of the fifth exon (Figure 1C). RT–PCR and sequencing revealed that $NIL^{H12\text{-}29}$ contains only a single transcript, whereas NIL^{FH212} harbors three transcript variants (Supplemental Figure 4A). Compared with the cDNA from H12-29 and Nipponbare, *TGW5-FH1* (the first transcript variant from FH212) contains a 19-bp insertion and a 13-bp deletion at the splicing site, whereas *TGW5-FH2* harbors only the 13-bp deletion, making the two variants indistinguishable by agarose electrophoresis. *TGW5-FH3* retains the fourth intron and is thus larger in size (Supplemental Figure 4B). In terms of their amino acid sequences, both TGW5-FH2 and TGW5-FH3 show premature termination, but the TGW5-FH1 protein is only slightly modified, with four variant residues and a two-residue insertion that may impair a conserved alpha-helix structure in the protein (Supplemental Figures 4C–4E). The CRISPR–Cas9-derived *TGW5* mutants *crj-01* and *crj-40* in the FH212 background showed a more severe dwarf phenotype and smaller grains than FH212, indicating that $TGW5^{FH212}$ is partially functional (Supplemental Figure 5). Constructs $proTGW5::TGW5^{H12\text{-}29}$ and $proTGW5::TGW5^{FH212(T869A)}$ were introduced into NIL^{FH212} and fully rescued its plant height and seed size to $NIL^{H12\text{-}29}$ levels (Figures 1D and 1E). We therefore concluded that $TGW5^{FH212}$ is a new weak allele of *D1*. The A869T SNP in $TGW5^{FH212}$ impairs mRNA splicing to produce a partially functional protein, TGW5-FH1, leading to a mild dwarf phenotype and a more minor seed phenotype in NIL^{FH212}.

Given the significant effect of recessive $TGW5^{FH212}$ on seed size, we developed two S-MSLs, S-C815S and S-WXS, by backcross

Published by the Plant Communications Shanghai Editorial Office in association with Cell Press, an imprint of Elsevier Inc., on behalf of CSPB and CEMPS, CAS.

This is an open access article under the CC BY license (http://creativecommons.org/licenses/by/4.0/).

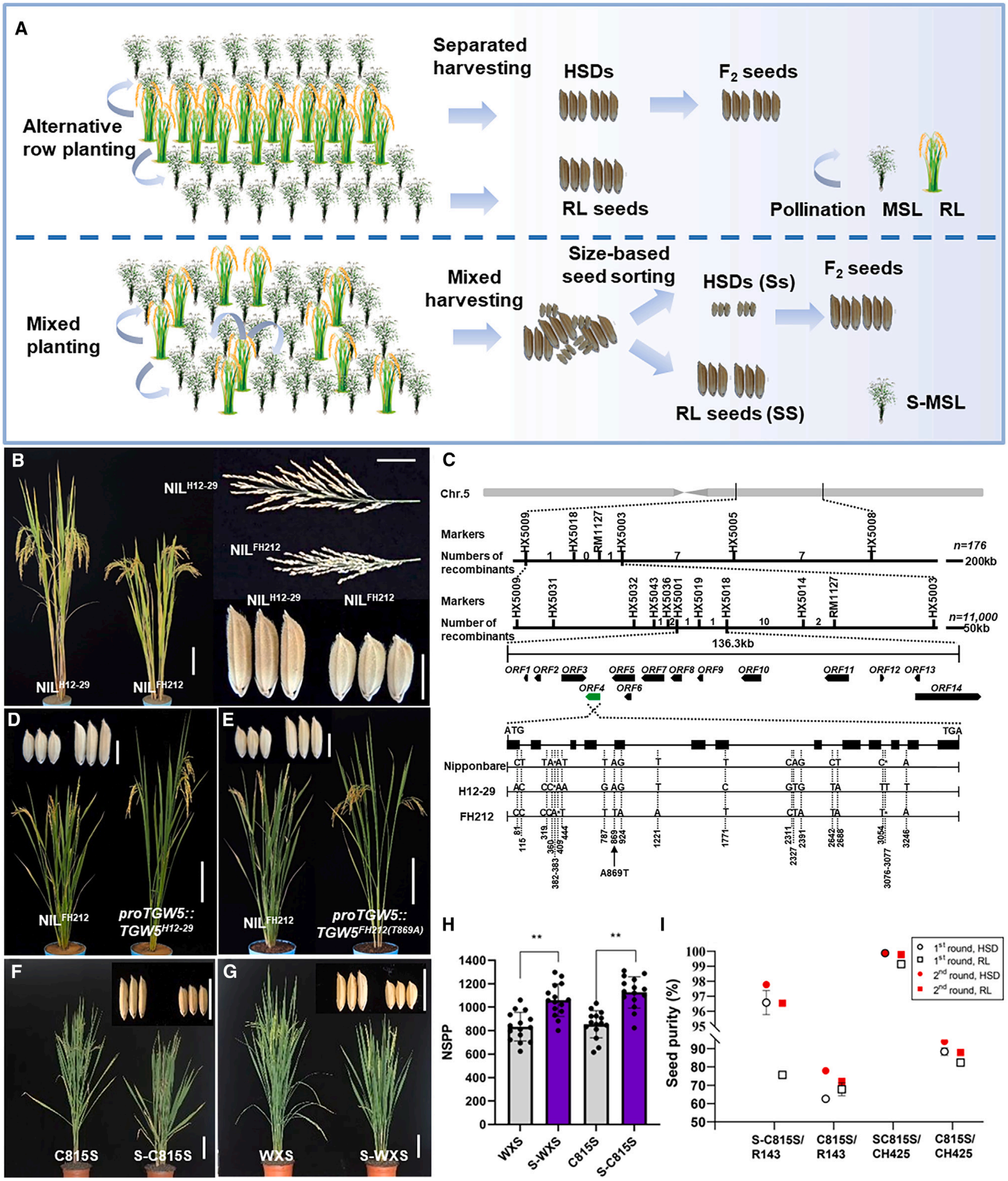

Figure 1. Cloning of *TGW5*, which controls grain size, and its use in mechanized production of rice hybrid seeds.
(A) Flow model of HSD production using conventional alternative-row planting and S-MSL-based mixed-planting strategies.
(B) Plant, panicle, and grain morphologies of the NIL lines. Plant, panicle, and grain scale bars represent 20 cm, 5 cm, and 5 mm, respectively.
(C) Map-based cloning and molecular characterization of *TGW5*.
(D and E) A genetic complementation test using *proTGW5::TGW5*$^{H12\text{-}29}$ and *proTGW5::TGW5*$^{FH212(T869A)}$ constructs. Scale bars for plants and grains represent 20 cm and 5 mm, respectively.

(legend continued on next page)

breeding in two elite temperature-sensitive MSLs, C815S and WuxiangS (WXS), respectively (Figures 1F and 1G; Supplemental Figure 6A). In terms of key MSL features, the S-MSLs showed fertility transition temperatures similar to those of their corresponding MSLs (Supplemental Table 1) but a ~20% reduction in stigma exertion rate compared with the MSLs (Supplemental Figures 6B–6D). At a fertile temperature, the S-MSLs had lower GL and TGW but slightly higher GW than their corresponding MSLs (Supplemental Figure 2). Notably, despite their somewhat lower absolute yield by weight, S-C815S and S-WXS set 31.31% and 27.82% more seeds per plant than C815S and WXS, respectively, mainly owing to their increased panicle numbers and spikelet density per panicle (Figure 1H; Supplemental Figures 2B–2D).

We next grew C815S and S-C815S at a sterile temperature and crossed them with the elite RLs R143 and CH425, respectively, to produce HSDs under agricultural conditions. The seed setting rates of C815S/CH425 and S-C815S/CH425 were 45.03% ± 3.04% and 38.15% ± 2.95%, respectively. Similarly, the seed setting rate of S-C815S/R143 (36.72% ± 2.78%) was slightly lower than that of C815S/R143 (40.75% ± 3.72%), possibly because of the lower stigma exertion rate of the S-MSLs. Interestingly, S-C815S/R143 and S-C815S/CH425 produced 12.79% and 10.84% more seeds than C815S/R143 and C815S/CH425, respectively, suggesting that the S-MSLs enable significantly higher seed propagation than the corresponding MSLs (Supplemental Figure 2C). An analysis of GL distribution indicated that the significant reduction of GL conferred by *TGW5*^{FH212} makes the S-MSL-derived HSDs separable from the RL seeds (Supplemental Figure 7). Indeed, S-C815S/CH425 HSDs (GL 6.58 ± 0.04 mm) and CH425 seeds (GL 10.72 ± 0.06 mm) could easily be sorted to reach an HSD purity of 99.87 ± 0.06% by one round of sorting with industrial alveolar cylinder equipment (Figure 1I; Supplemental Video 1). Moreover, the S-C815S/R143 HSDs (GL 6.61 ± 0.04 mm) and R143 seeds (GL 9.91 ± 0.05 mm) could also be effectively sorted to reach 96.56% ± 0.80% HSD seed purity (Figure 1I; Supplemental Video 2). By contrast, the regular MSL-derived HSDs and their corresponding RLs were not well separated, even after two rounds of sorting (Figure 1I; Supplemental Videos 3 and 4). This result clearly demonstrated that effective seed sorting of S-MSL-derived HSDs from RL seeds is highly feasible.

Finally, we evaluated the major agronomic traits of the F_1 plants of C815S/R143, S-C815S/R143, C815S/CH425, and S-C815S/CH425 in the paddy field. The hybrid lines derived from MSLs and from their corresponding S-MSLs had almost identical phenotypes, indicating that the use of *TGW5* in the S-MSLs had no negative effect on heterosis of the F_1 plants (Supplemental Figure 2).

In conclusion, we cloned a complete recessive seed size gene, *TGW5*^{FH212,} that encodes a weak allele of *D1*. Introduction of *TGW5*^{FH212} converted regular MSLs into S-MSLs with increased seed numbers and reduced seed size but no penalties in terms of F_1 heterosis. Although the trade-off between seed size and seed number is unfavorable for yield in the breeding practice of inbred lines, the smaller seed size and greater seed numbers conferred by *TGW5*^{FH212} turn out to be triple bonuses for HSD production: (1) they enable production of more HSDs per plant; (2) they facilitate effective post-harvest, size-based seed sorting that is compatible with labor-saving mechanized cultivation, which is estimated to reduce field costs by 16.3% (Tang et al., 2020); and (3) they reduce HSD storage and transport costs because of the smaller HSD seed size. Given that the currently used commercial MSLs and RLs have very similar seed sizes, *TGW5*^{FH212} is a highly promising resource for S-MSL breeding and is expected to increase HSD propagation and cut the cost of HSD production through mechanized cultivation.

SUPPLEMENTAL INFORMATION

Supplemental information is available at *Plant Communications Online*.

FUNDING

This work was supported by the National Natural Science Foundation of China (grant nos. 32072050, U22A20456, and U20A2030), a special support plan for high-level talents in Zhejiang (grant no. 2022R52020), the Zhejiang Provincial Natural Science Foundation of China (grant no. LZ21C130001), the National Key R&D Program of China (2020YFE0202300), Open Project Funding of the State Key Laboratory of Biocatalysis and Enzyme Engineering, the CNRRI key research and development project (CNRRI-2020-01), and the ASTIP program of CAAS.

AUTHOR CONTRIBUTIONS

J.Z., J.Y., and X.S. planned and designed the research; Y.Q., J.Y., F.Z., L.D., P.C., M.Y., Y.W., X.T., J.H., and Z.L. performed experiments; J.Z., Y.Q., J.Y., and F.Z. analyzed data; and J.Z. and J.Y. wrote the manuscript.

ACKNOWLEDGMENTS

The authors thank the research group of gene mapping and cloning, CNRRI for supplying the rice material of H12-29 and the support in TGW5 cloning and breeding of S-MSLs, Prof. Yajing Guan from Zhejiang University, Mr. He Zhang from Shenghong Planting & Seedling Co., Ltd., and Dr. Sanqiang Zhang from Hubei University of Technology for assisting with HSD sorting using the alveolar cylinder and Prof. Tingxu Huang (Institute of Rice Research, Fujian Academy of Agricultural Sciences) for providing the parent line Fuhui212. No conflict of interest is declared.

Received: December 18, 2023
Revised: December 27, 2023
Accepted: January 4, 2024
Published: January 11, 2024

Jiezheng Ying[1,4], Yaobing Qin[1,4], Fengyong Zhang[1,4], Liu Duan[2], Peng Cheng[3], Man Yin[1], Yifeng Wang[1], Xiaohong Tong[1], Jie Huang[1], Zhiyong Li[1], Xianjun Song[3] and Jian Zhang[1,]*

(F and G) Plant and grain morphologies of C815S and S-C815S **(F)** and WXS and S-WXS **(G)**. Scale bars for plants and grains represent 12 cm and 10 mm, respectively.
(H) Number of seeds per plant in the HSD production experiments. MSLs and S-MSLs were sterile and pollinated by the RL lines. **$P < 0.01$, Student's *t*-test.
(I) Seed purities of various seeds after sorting with an alveolar cylinder.

[1]State Key Lab of Rice Biology and Breeding, China National Rice Research Institute, Hangzhou 311400, China
[2]State Key Laboratory of Biocatalysis and Enzyme Engineering, School of Life Sciences, Hubei University, Wuhan 430062, China
[3]Key Laboratory of Plant Molecular Physiology, Institute of Botany, the Chinese Academy of Sciences, Beijing 100093, China
[4]These authors contributed equally to this article.
*Correspondence: Jian Zhang (zhangjian@caas.cn)
https://doi.org/10.1016/j.xplc.2024.100811

REFERENCES

Cheng, S.H., Zhuang, J.Y., Fan, Y.Y., Du, J.H., and Cao, L.Y. (2007). Progress in research and development on hybrid rice: a super-domesticate in China. Ann. Bot. **100**:959–966.

Dai, J. (1996). Recent research on hybrid rice in Japan. Hybrid. Rice **6**:37.

Fu, Y., Zhu, Z., Xiao, H., Hu, G., Si, H., Yu, Y., and Sun, Z. (2001). Primary Study on Mechanization of Seed Production of Hybrid Rice by Inducing Bar Gene to Peiai 64S. Chin. J. Rice Sci. **15**:97–100.

Xuedan, L., Fan, L., Yunhua, X., Feng, W., Guilian, Z., Huabing, D., and Wenbang, T. (2023). Grain Shape-Genes: Shaping the Future of Rice Breeding. Rice Sci. **30**:379–404.

Maruyama, K., Kato, H., and Araki, H. (1991). Mechanized production of F1 seeds in rice by mixed planting. Jpn. Agric. Res. Q. **24**:243–252.

Oki, K., Inaba, N., Kitano, H., Takahashi, S., Fujisawa, Y., Kato, H., and Iwasaki, Y. (2009). Study of novel d1 alleles, defective mutants of the alpha subunit of heterotrimeric G-protein in rice. Genes Genet. Syst. **84**:35–42.

Qin, Y., Cheng, P., Cheng, Y., et al. (2018). QTL-seq identified a major QTL for grain length and weight in rice using near isogenic F2 population. Rice Sci. **25**:121–131.

Tang, W., Zhang, G., and Deng, H. (2020). Technology Exploration and Practice of Hybrid Rice Mechanized Seed Production. Chin. J. Rice Sci. **34**:95–103.

Wu, J., Qiu, S., Wang, M., Xu, C., Deng, X.W., and Tang, X. (2021). Construction of a weight-based seed sorting system for the third-generation hybrid rice. Rice **14**:66–67.

Xia, Y., Tang, N., Hu, Y., Li, D., Li, S., Bu, X., Yu, M., Qi, S., Yang, Y., Zhu, H., et al. (2019). A method for mechanized hybrid rice seed production using female sterile rice. Rice **12**:39.

 © 2024 The Author(s).

Plant Communications, Volume *5*

Supplemental information

A weak allele of *TGW5* enables greater seed propagation and efficient size-based seed sorting for hybrid rice production

Jiezheng Ying, Yaobing Qin, Fengyong Zhang, Liu Duan, Peng Cheng, Man Yin, Yifeng Wang, Xiaohong Tong, Jie Huang, Zhiyong Li, Xianjun Song, and Jian Zhang

Materials and Methods

Plant materials and field trials

To identify the *TGW5* gene, a large grain indica variety Hui 12-29 (H12-29) was crossed with a small grain *indica* variety Fuhui 212 (FH212) to develop advanced genetic populations. Recombinant plants in the F_8 generation with identical backgrounds but heterozygous *TGW5* region were selected to develop segregating populations for fine mapping. From one recombinant plant in F_{10} generation, we developed a pair of nearly isogenic lines for *TGW5*, $NIL^{H12-29}$and NIL^{FH212}, with isogenic homozygous background and a small homozygous chromosomal segment of H12-29 and FH212 containing *TGW5*, respectively. To develop thermo-sensitive genic male sterile lines with small grain size, NIL^{FH212} was crossed with two commercial thermo-sensitive male sterile lines, C815S and WuxiangS. After successive backcrosses with C815S and WuxiangS and marker assistant selections, two thermo-sensitive male sterile lines with small grain size in BC_3F_5 generation, S-C815S and S-WXS, were developed and characterized by *de novo* genome sequencing.

Rice materials were planted with a spacing of 16.6 cm × 26.7 cm in the experimental stations of China National Rice Research Institute in Hangzhou (119°57' E, 30°05' N) and Lingshui (109°45' E, 18°22' N). Eighteen plants for each line were planted in 3 rows, and the 4 plants in the middle of the row were harvested for agronomic traits characterization. For HSD production, MSLs and RLs were grown in separate rows with a ratio of 7:2 (every 7 rows of MSLs neighbored by 2 rows of RLs). Field management followed the local agricultural practice in production.

Fine mapping and candidate gene analysis

Segregating populations containing about 11,000 plants in the F_9 generation were used to screen the recombinants in the target region around *TGW5*. We developed molecular markers based on 30× genome re-sequencing of the parental lines and detected 17 recombinants in the target region containing *TGW5*. The F_{10} progeny

derived from the recombinant plants were used to screen homozygous recombination products and measure the grain size, including grain length, width, and 1000-grain weight. The candidate *TGW5* genes from H12-29 and FH212 genomic DNA were PCR amplified and sequenced. The sequences of the primers are provided in Table S2.

RNA extraction and cDNA isolation

Total RNA was extracted from various tissues of the parental lines, H12-29 and FH212, using RNeasy Plant Mini Kit (QIAGEN, Germantown, MD, USA) and was converted into cDNA with ReverTra Ace qPCR RT Master Mix with gDNA Remover (TOYOBO, Shanghai, China). Full-length *TGW5* cDNA was amplified from the first-strand cDNA with KOD-Plus-Neo Kit (TOYOBO, Shanghai, China) and sequenced.

Vector construction and transgenic analysis

We developed EH2 primers with restriction enzyme cleavage sites of *Eco*R I and *Hin*d III gDNA to amplify gDNA sequences of *TGW5* containing 2,000 bp promoter regions, full-length ORFs, and 500 bp terminator sequences from H12-29 and FH212 genomic DNA using KOD FX Neo (TOYOBO, Shanghai, China), respectively. Both *TGW5* gDNA sequences were sub-cloned into the vector pCambia 1301. We further developed XD1 primers to create SNP mutation T869A in the *TGW5* gDNA sequence of FH212 in the pCambia 1301 using the Fast Mutagenesis System (TransGen, Beijing, China). *TGW5* gene in FH212 was knocked out using the CRISPR/Cas9 system according to a previously described protocol. The constructs containing the *TGW5* gDNA sequence of H12-29 and the T869A point mutation *TGW5* gDNA sequence of FH212 were used for the complementation test and mutation validation. The resulting constructs were introduced into *Agrobacterium tumefaciens* strain EHA105 and transferred into NIL^{FH212}. The sequences of the primers are provided in Table S2.

Measurement of agronomic traits

Rice grains collected from the paddy field were first air-dried, then dried at 37°C in the oven for 24 hours. Four traits, including plant height, number of panicles per plant (NPP), seeds per plant, grain yield per plant, and stigma exertion rate were measured manually. The filled grains were chosen to identify the grain traits, including grain length (GL), grain width (GW), and 1000-grain weight (TGW) using an SC-A seed counting and grain weighting device (Wanshen Ltd, Hangzhou, China).

Histological observation

To determine the cell size, cell length, and cell width, spikelet hulls of mature grain of NILs (*TGW5*) and transgenic plants were sputter-coated with platinum and observed using a scanning electron microscope (S-4800; Hitachi, Japan). Cell size, length, width, and cell number in the outer parenchyma layer of the spikelet hulls were analyzed using ImageJ software.

Protein sequence analysis

Multiple alignments of amino acid sequences of TGW5 were conducted online (https://www.ebi.zc.uk/Tools/msa/clustalo) and were described using the Sequence Manipulation Suite (Li et al., 2016). SWISS-MODEL was used in the homology modeling of TGW5 protein conformation. Protein structures corresponding to different transcripts of the *TGW5* gene were analyzed with Swiss-Pdb Viewer.

GL-based seed sorting

The HSD and RL seeds were mixed and sorted through an alveolar cylinder (Westrup LA-T, Denmark) with an alveolar diameter of 8.5 mm and a cylinder rotating speed in level 1. The vibration frequency was set to level 1 for S-C815S/R143 sorting and level 2 for the other samples.

Figure S1. Comparison of the rice stem length between the NILs. (A) The internodes of NIL^{H12-29} and NIL^{FH212}. Bar, 20 cm. (B) Internode lengths relative to the total length of the stem. (C) The percentage of internode length relative to the total internode length. White arrows indicate the nodes.

Figure S2. The major agronomic traits of the genetic materials.
(A) Plant height. (B) Panicles per plant. (C) Seeds per plant. (D) Grain yield per plant. (E) Grain length. (F) Grain width. (G) Thousand-grain weight. For data in grey, pink, and purple bars, plants were grown in Hangzhou, Zhejiang, China (119°57' E, 30°05' N), where the average temperature was above 25℃ during the early panicle differentiation stage at the end of July 2023. All the MSLs were sterile and cross-pollinated by RLs, while inbred lines were fully fertile. For data in orange and green bars, plants were grown in Lingshui, Hainan, China (109°45' E, 18°22' N), where the average temperature was below 22℃ during the early panicle differentiation stage in the middle of February 2023. All the MSLs were fertile and self-pollinated. *, $P<0.05$; **, $P<0.01$ by *students*' t-test.

Figure S3. Cytological feature observations of exterior epidermal cells of the spikelet hulls of NIL^{H12-29} and NIL^{FH212}. (A-B) Exterior epidermal cells of the lemma of NIL^{H12-29} (A) and NIL^{FH212} (B), Bar, 100μm. (C) Comparison of cell length of the spikelet hulls of NIL^{H12-29} and NIL^{FH212}. (D) Comparison of cell width of the spikelet hulls of NIL^{H12-29} and NIL^{FH212}. (E) Comparison of cell size of the spikelet hulls of NIL^{H12-29} and NIL^{FH212}. Scale bar, 100 μm. *, P＜0.05, ****, P＜0.0001.

Figure S4. Structural and sequence analysis of *TGW5*. (A) Electrophoresis of *TGW5* gDNA and cDNA PCR products from the parental lines H12-29 and FH212, FH1, FH2, and FH3 are the three transcripts of $TGW5^{FH212}$. (B) Schematic presentation of the variations on *TGW5* transcript. (C) Alignment of cDNA sequences of TGW5-FH variants. TGW5-FH2 and TGW5-FH3 are pre-matured at position 574 and 352, respectively. (D) Alignment of amino acid sequences of TGW5 between H12-29 and TGW5-FH1. Red box, amino acid variations for the impaired alpha helix structure in FH1. (E) Predicted three-dimensional structure of TGW5 based on the deduced amino acid sequence. The red circle indicates the mutation site of TGW5-FH1, potentially resulting in an impaired alpha helix structure.

Figure S5. Phenotypes and genotypes of CRISPR/Cas9-derived *TGW5* knock-out lines in FH212 background. (A-C) Plant (A), grain (B), and panicle (C) morphologies of wild-type FH212 and gene-edited lines. Bar, 10 cm in A, 1 cm in B, and 2 cm in C. (D) Sequence alignment of the targeted mutation site in *TGW5*. The PAM sequences are highlighted in yellow. - indicates base deletion; Red font indicates base insertions.

Figure S6. Schematic presentation of the genetic backgrounds and stigma exertion features of S-C815S and S-WXS.

(A) Genome *de novo* sequencing results showed that there are 5.71% and 4.78% of the donor chromosome segments in S-C815S (BC_3F_5) and S-WXS (BC_3F_5), respectively. The black bar indicates the genome fragment from donor FH212. (B-C) The stigma exertion features of C815S (B) and S-C815S (C). The embedded pictures on the bottom depict a zoomed-in view of the white frame at the top. The red triangle indicates florets with fully exerted stigmas. Scale bars, 5 mm. (J) Total percentage of the exerted stigma (TPES). ** indicates $P<0.01$ by *students*' t-test, n=50.

Figure S7. The grain length distribution frequencies of HSDs and RLs.

(A) The grain length distribution of R143 and HSDs. (B) The grain length distribution of CH425 and HSDs. All the data were randomly collected from over 500 grains of each line.

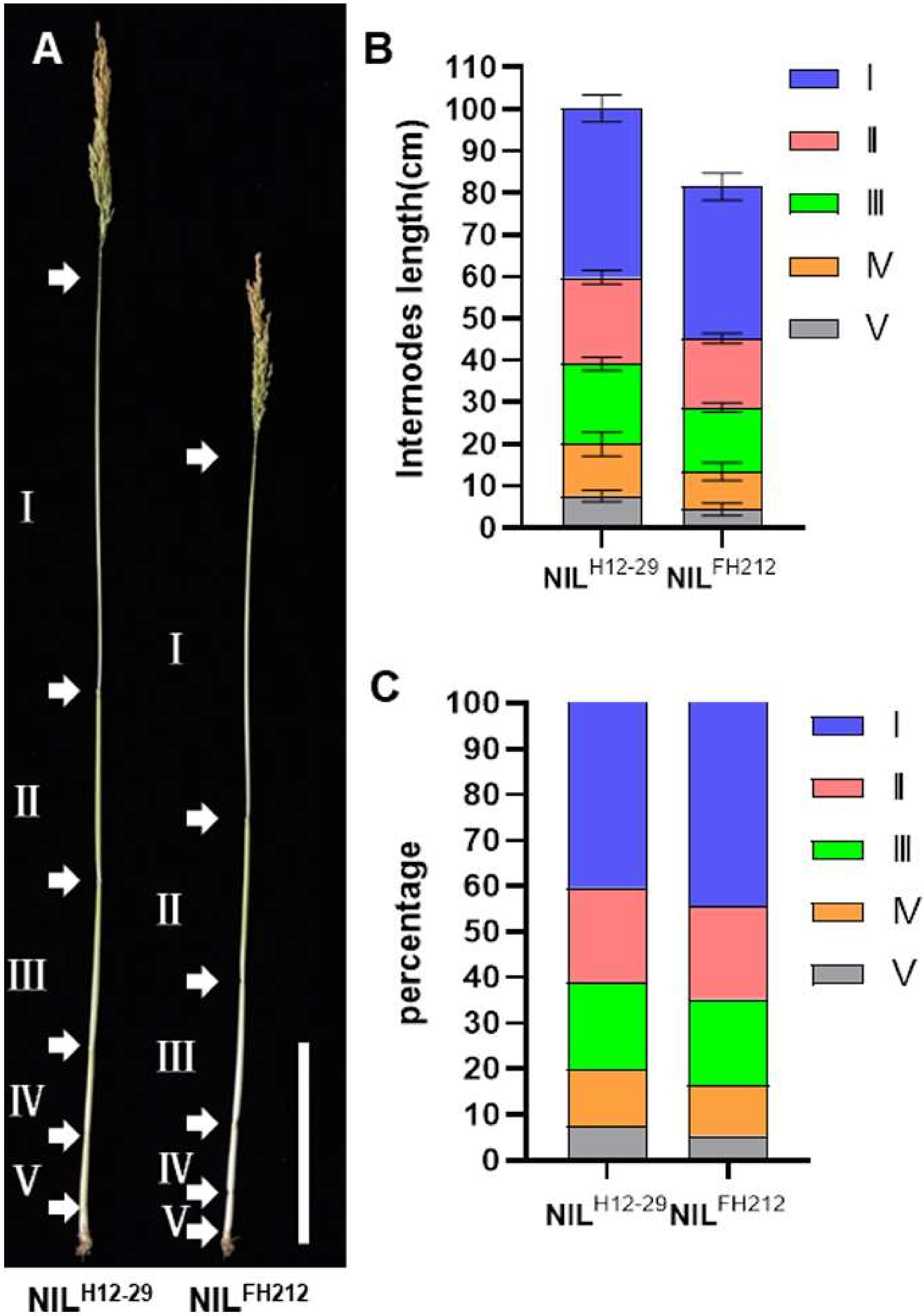
A
I
II
III
IV
V
I
II
III
IV
V
NIL^H12-29
NIL^FH212
B
Internodes length(cm)
110
100
90
80
70
60
50
40
30
20
10
0
NIL^H12-29
NIL^FH212
I
II
III
IV
V
C
percentage
100
90
80
70
60
50
40
30
20
10
0
NIL^H12-29
NIL^FH212
I
II
III
IV
V

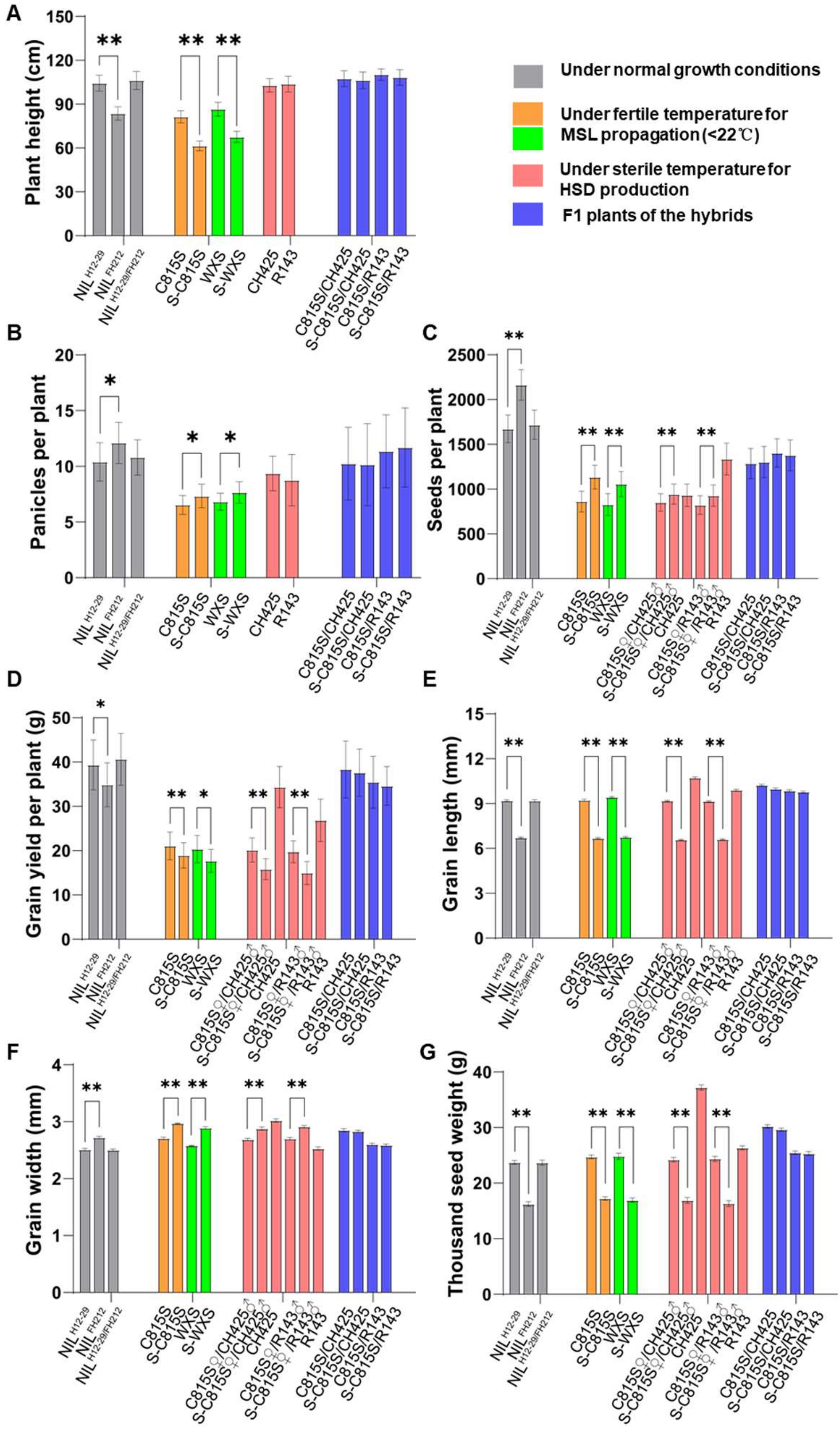

A
B
C
D
E
F
G
Plant height (cm)
Panicles per plant
Seeds per plant
Grain yield per plant (g)
Grain length (mm)
Grain width (mm)
Thousand seed weight (g)
Under normal growth conditions
Under fertile temperature for MSL propagation (<22℃)
Under sterile temperature for HSD production
F1 plants of the hybrids
C815S
S-C815S
WXS
S-WXS
CH425
R143
C815S/CH425
S-C815S/CH425
C815S/R143
S-C815S/R143

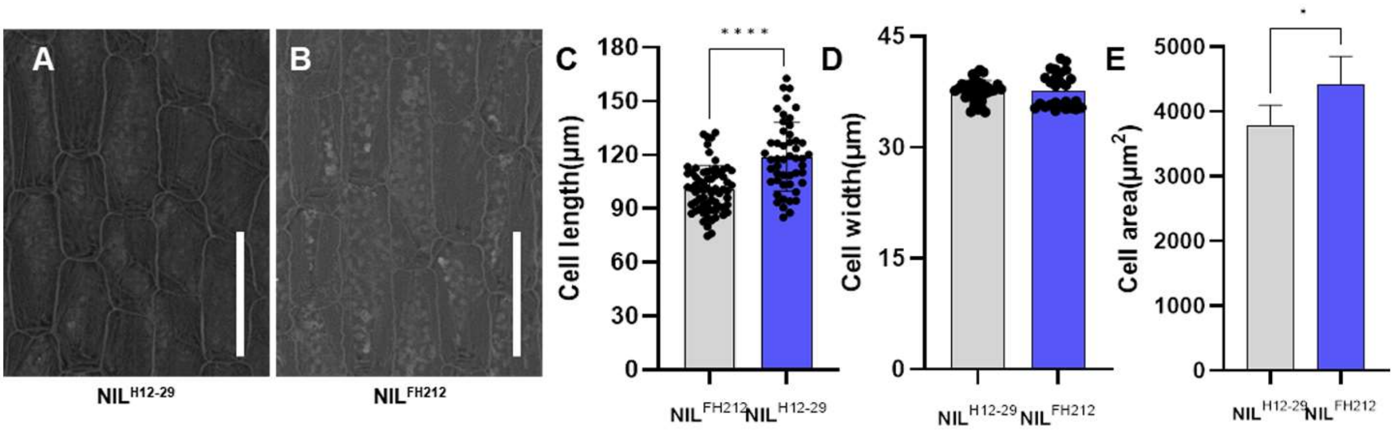

A
B
NIL^H12-29
NIL^FH212
C
Cell length(μm)
****
180
150
120
90
60
30
0
NIL^FH212 NIL^H12-29
D
Cell width(μm)
45
30
15
0
NIL^H12-29 NIL^FH212
E
Cell area(μm²)
*
5000
4000
3000
2000
1000
0
NIL^H12-29 NIL^FH212

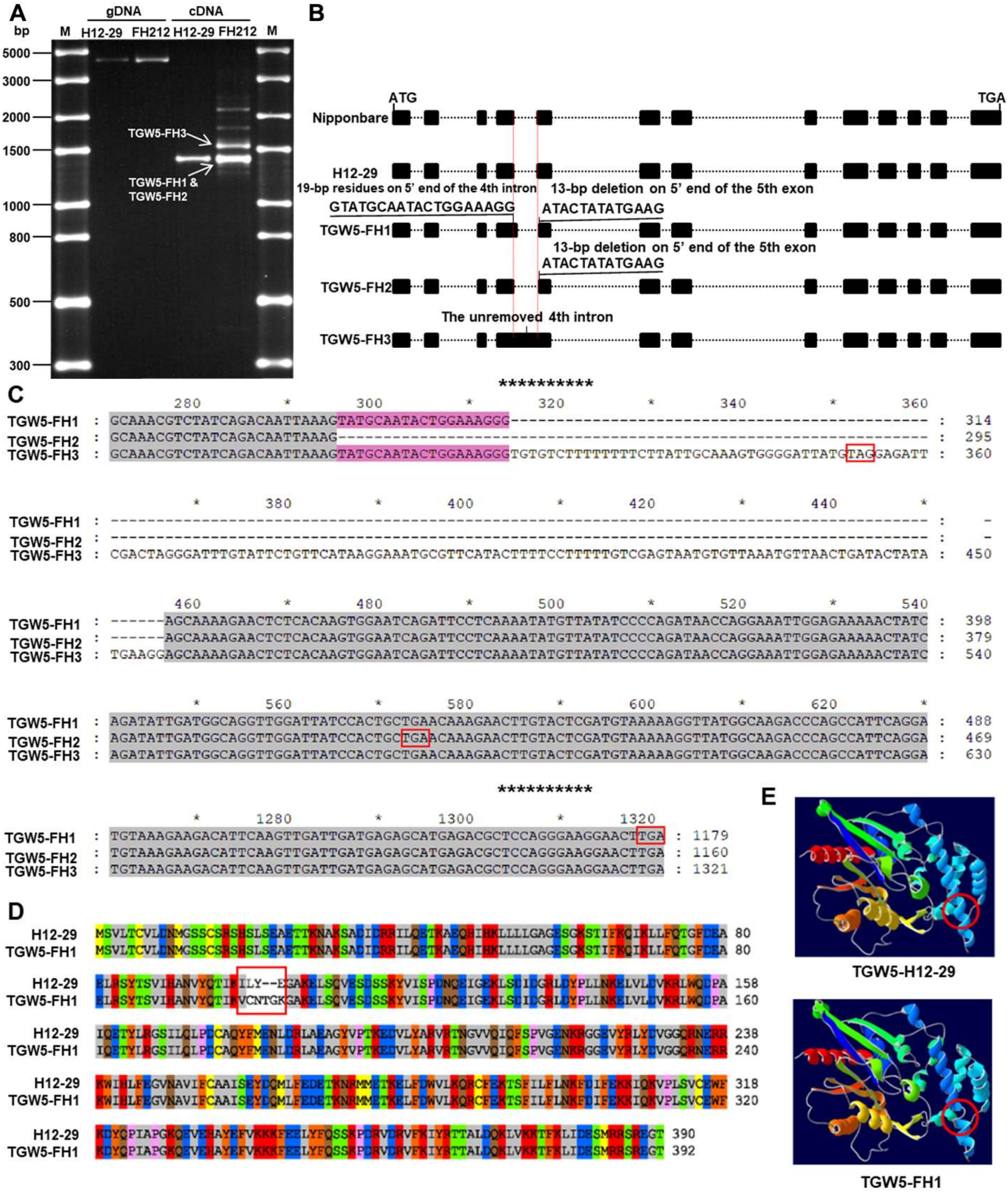

A
bp
M
gDNA
cDNA
H12-29
FH212
H12-29
FH212
M
5000
3000
2000
1500
1000
800
500
300
TGW5-FH3
TGW5-FH1 &
TGW5-FH2
B
ATG
TGA
Nipponbare
H12-29
19-bp residues on 5' end of the 4th intron
13-bp deletion on 5' end of the 5th exon
GTATGCAATACTGGAAAGG
ATACTATATGAAG
TGW5-FH1
13-bp deletion on 5' end of the 5th exon
ATACTATATGAAG
TGW5-FH2
The unremoved 4th intron
TGW5-FH3
C
TGW5-FH1 : GCAAACGTCTATCAGACAATTAAAGTATGCAATACTGGAAAGG : 314
TGW5-FH2 : GCAAACGTCTATCAGACAATTAAAG : 295
TGW5-FH3 : GCAAACGTCTATCAGACAATTAAAGTATGCAATACTGGAAAGGTGTGTCTTTTTTTTCTTATTGCAAAGTGGGGATTATGTAGGAGATT : 360
TGW5-FH3 : CGACTAGGGATTTGTATTCTGTTCATAAGGAAATGCGTTCATACTTTTCCTTTTTGTCGAGTAATGTGTTAAATGTTAACTGATACTATA : 450
TGW5-FH1 : AGCAAAAGAACTCTCACAAGTGGAATCAGATTCCTCAAAATATGTTATATCCCCAGATAACCAGGAAATTGGAGAAAAACTATC : 398
TGW5-FH2 : AGCAAAAGAACTCTCACAAGTGGAATCAGATTCCTCAAAATATGTTATATCCCCAGATAACCAGGAAATTGGAGAAAAACTATC : 379
TGW5-FH3 : TGAAGGAGCAAAAGAACTCTCACAAGTGGAATCAGATTCCTCAAAATATGTTATATCCCCAGATAACCAGGAAATTGGAGAAAAACTATC : 540
TGW5-FH1 : AGATATTGATGGCAGGTTGGATTATCCACTGCTGAACAAAGAACTTGTACTCGATGTAAAAAGGTTATGGCAAGACCCAGCCATTCAGGA : 488
TGW5-FH2 : AGATATTGATGGCAGGTTGGATTATCCACTGCTGAACAAAGAACTTGTACTCGATGTAAAAAGGTTATGGCAAGACCCAGCCATTCAGGA : 469
TGW5-FH3 : AGATATTGATGGCAGGTTGGATTATCCACTGCTGAACAAAGAACTTGTACTCGATGTAAAAAGGTTATGGCAAGACCCAGCCATTCAGGA : 630
TGW5-FH1 : TGTAAAGAAGACATTCAAGTTGATTGATGAGAGCATGAGACGCTCCAGGGAAGGAACTTGA : 1179
TGW5-FH2 : TGTAAAGAAGACATTCAAGTTGATTGATGAGAGCATGAGACGCTCCAGGGAAGGAACTTGA : 1160
TGW5-FH3 : TGTAAAGAAGACATTCAAGTTGATTGATGAGAGCATGAGACGCTCCAGGGAAGGAACTTGA : 1321
D
H12-29
TGW5-FH1
E
TGW5-H12-29
TGW5-FH1

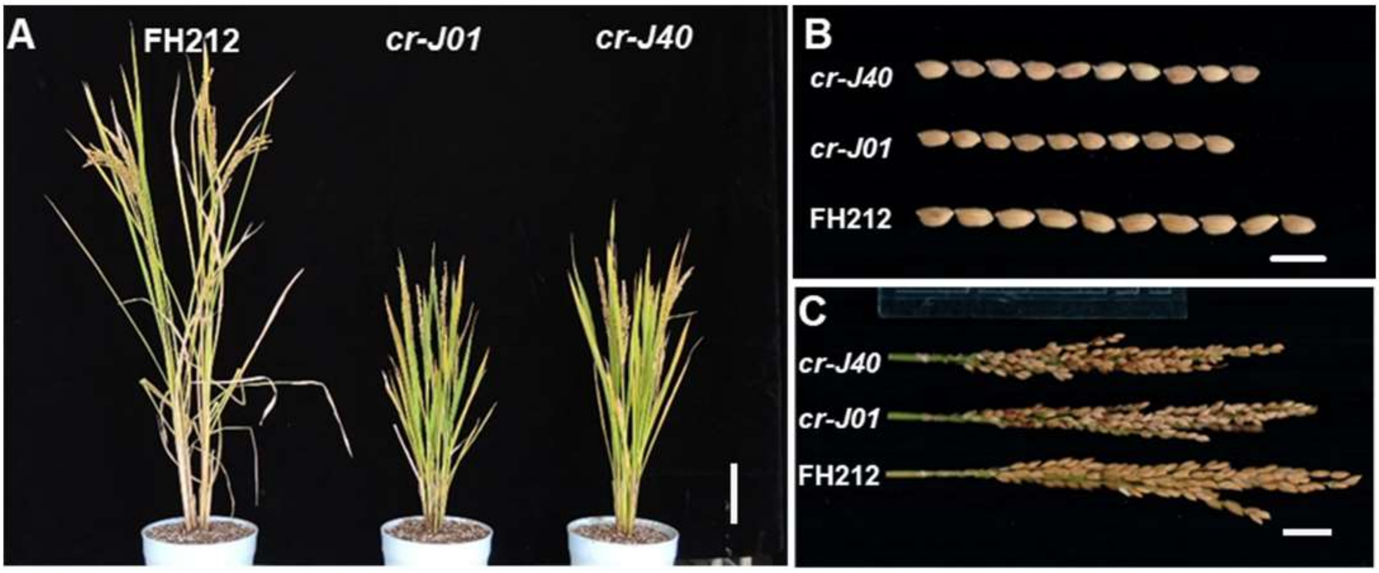

D

FH212 TAGGAGGCCAGAGGAATGAGAGGAGAAAGTGGATTCATCTTTTTGA

Cr-J01 TAGGAGGCCAGAGGAATGAGAG- - GAAAGTGGATTCATCTTTTTGA

Cr-J40 TAGGAGGCCAGAGGAATGAGAGGAGACACAAAAAGTGGATTCATCTTTTTGA

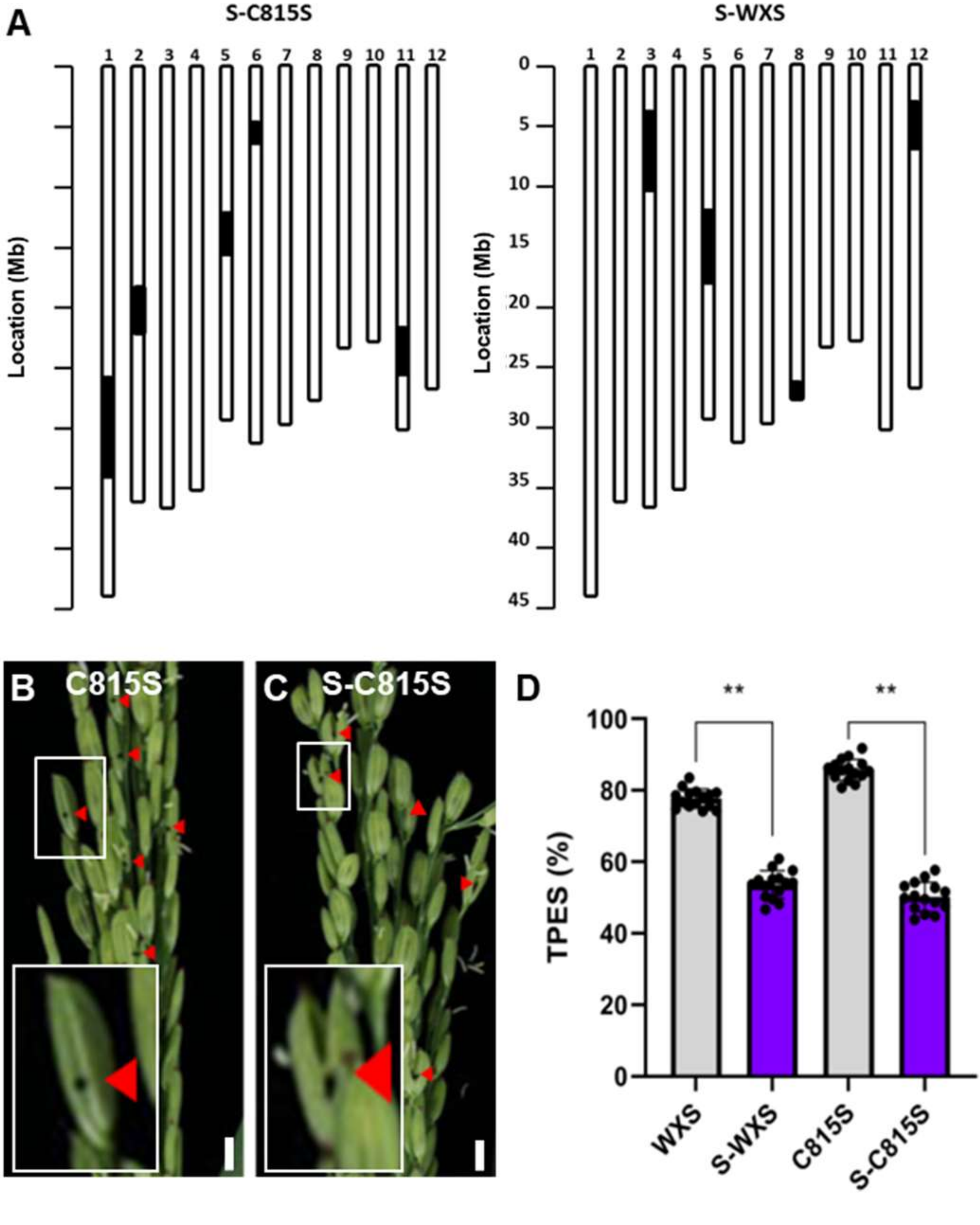
A
S-C815S
S-WXS
Location (Mb)
0
5
10
15
20
25
30
35
40
45
1
2
3
4
5
6
7
8
9
10
11
12
B
C815S
C
S-C815S
D
TPES (%)
**
0
20
40
60
80
100
WXS
S-WXS
C815S
S-C815S

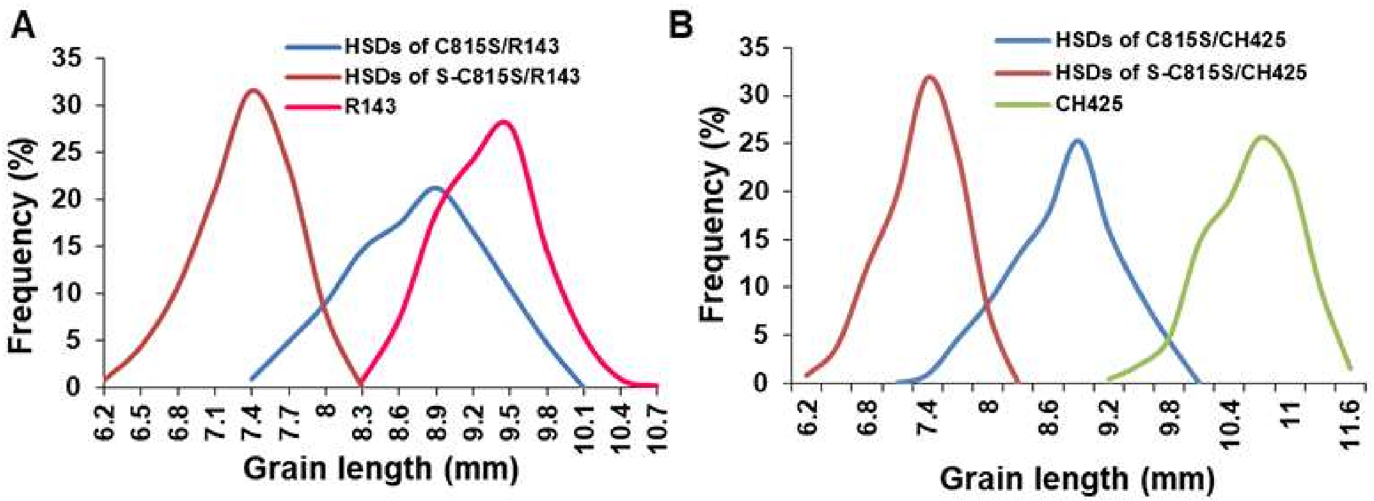
A
HSDs of C815S/R143
HSDs of S-C815S/R143
R143
Frequency (%)
Grain length (mm)
B
HSDs of C815S/CH425
HSDs of S-C815S/CH425
CH425
Frequency (%)
Grain length (mm)
